# Molecular detection, serotyping, cytotoxicity, and antimicrobial resistance of STEC and EPEC isolated from milk and milk products in northern India

**DOI:** 10.3389/fmicb.2026.1748367

**Published:** 2026-02-18

**Authors:** Jubeda Begum, Shubhangi Nigam, Nasir Akbar Mir

**Affiliations:** 1 College of Veterinary and Animal Sciences, Govind Ballabh Pant University of Agriculture and Technology, Pantnagar, India; 2 Veterinary Technology, Higher Education Department, Jammu and Kashmir, India

**Keywords:** antibiotic resistance, EPEC, milk, serotyping, STEC, Vero cell cytotoxicity, virulence genes

## Abstract

Shiga toxin-producing *Escherichia coli* (STEC) and Enteropathogenic *E. coli* (EPEC) are important foodborne pathogens posing significant public health threats. This cross-sectional study investigated the prevalence, virulence profiles, serotypes, cytotoxicity, and antimicrobial resistance profiles of STEC and EPEC from milk and milk products in Uttarakhand, Northern India. A total of 680 samples (260 raw milk and 420 milk product samples) were collected from dairy farms, milk shops, collection centers, and street vendors over 9 months and screened for *E. coli* using conventional and molecular methods. Multiplex PCR targeting *stx1, stx2, eaeA,* and *hlyA* genes was employed to identify virulent isolates, which were further serotyped, evaluated for cytotoxicity on Vero cells, and tested for antibiotic susceptibility against 19 antibiotics. Resistant isolates were screened for *tetA, tetB, sul1,* and *CITM* genes by PCR. *E. coli* was detected in 28.82% of samples, with higher prevalence in raw milk (31.15%) than milk products (27.38%). Among isolates, 39.8% harbored at least one virulence gene, with *stx1* being most prevalent. Serotyping revealed 22 O-serogroups, predominantly O18, O111, O120, O126, and O17. All *stx*-positive isolates showed cytopathic effects in Vero cells, enhanced after ciprofloxacin induction. High resistance was observed against ampicillin, tetracycline, oxytetracycline, cephalothin, and sulphonamides, while imipenem, gentamicin, and nalidixic acid were most effective. Among multidrug-resistant isolates, 95% carried *tetB, sul1,* or *CITM* genes, while *tetA* was absent. The study confirms the presence of virulent and multidrug-resistant STEC and EPEC in milk and dairy products, highlighting the need for improved hygiene, judicious antimicrobial use, and regular monitoring to mitigate food safety and zoonotic risks.

## 1 Introduction

India is the world’s largest producer of milk, with an annual output of 221.06 million tons and a per capita availability of 444 g per day (IBEF Report, 2023). However, only 25–30% of this milk is processed by the organized sector, while the majority is sold through unregulated channels, predisposing it to microbial contamination (Pattanaik and Samal, 2013). *Escherichia coli*, a commensal organism of the intestinal tract in humans and animals, frequently enters milk and milk products through fecal contamination during milking, handling, or processing under suboptimal unhygienic conditions (Shahrivar et al., 2025; Chowdhury et al., 2024). Although most *E. coli* strains are harmless, some acquire virulence genes and become pathogenic, leading to enteric and systemic diseases in both animals and humans (Begum et al., 2018; Chowdhury et al., 2024).

Among the pathogenic variants, Shiga toxin-producing *E. coli* (STEC) and enteropathogenic *E. coli* (EPEC) are of particular public health concern. These pathotypes carry key virulence genes encoding Shiga toxins (*stx1, stx2*), intimin (*eaeA*), and hemolysin (*hlyA*) (Ranjbar et al., 2018; Dutta et al., 2011; Ombarak et al., 2016). Shiga toxins and hemolysin are responsible for tissue damage, while intimin mediates intimate attachment and effacing lesions on intestinal epithelial cells. STEC infections are a major cause of foodborne illness worldwide, leading to hemorrhagic colitis and hemolytic uremic syndrome (Schallan et al., 2011; Rafee et al., 2025).

In addition to virulence, the emergence of antimicrobial resistance (AMR) in STEC and EPEC has become a serious global concern. These bacteria act as reservoirs and disseminators of antibiotic resistance genes across species (Dandachi et al., 2018; Begum and Mir, 2023; Ali et al., 2024), and resistant strains have been widely reported in milk and dairy products (de Verdier et al., 2012; Elmonir et al., 2021). The high resistance rates to ampicillin, tetracycline, sulfonamides, and cephalosporins are largely attributed to indiscriminate antibiotic usage in livestock production (Wang et al., 2016).

Furthermore, serotyping of *E. coli* isolates provides crucial epidemiological insights, as certain O-serogroups (e.g., O26, O111, O157) are strongly associated with virulent and zoonotic strains (Miko et al., 2014). Likewise, Vero cell cytotoxicity assays offer functional validation of Shiga toxin expression and are considered the gold standard for confirming phenotypic virulence (Xia et al., 2010; To and Bhunia, 2019).

Considering the limited data on the molecular characterization, serogroup distribution, virulence expression, and antimicrobial resistance of *E. coli* in dairy sources from Northern India, the present study aimed to bridge this gap through a multi-tiered assessment by investigate the prevalence, virulence profiles, serotypes, cytotoxicity, and resistance gene patterns of STEC and EPEC isolated from milk and milk products in Uttarakhand.

## 2 Materials and methods

### 2.1 Study design and sampling

A cross-sectional sampling design covering different distribution points was followed. A total of 260 raw milk samples and 420 milk product samples (Dahi/curd – 100, Lassi/sweet buttermilk – 100, Ghee/clarified butter – 100, and Paneer/Indian cottage cheese – 120) were collected from October 2021 to June 2022 The samples were collected from twelve dairy farms, eight milk retail shops, six milk collection centers, and six street vendors in Uttarakhand, India (Supplementary Table S1). The selection of sampling sites was done randomly and only one sample per site per visit was collected. The samples were transported to the laboratory under a cold chain for microbiological analysis.

### 2.2 Bacterial culture

#### 2.2.1 Pre-enrichment of collected milk and milk product samples

Samples of milk and lassi were used as such; paneer and dahi samples were homogenized, and ghee samples were pre-warmed at 45 °C before enrichment. For the liquid samples (milk, lassi, ghee, and dahi), 1 mL of each sample was placed in a test tube, followed by the addition of 5 mL of sterile buffered peptone water. The tubes were mixed thoroughly and incubated in a shaking incubator for (12–18) hrs at 37 °C for pre-enrichment. For paneer, 1 gm of homogenized paneer sample was aseptically transferred into a sterile test tube and blended with 5 mL of sterile buffered peptone water. The samples were incubated in a shaking incubator for (12–18) hrs at 37 °C for enrichment.

#### 2.2.2 Isolation and characterization of *Escherichia coli* isolates

A loopful of enriched milk and milk product samples was streaked directly on MacConkey Agar (Hi-Media, Mumbai, India) and incubated at 37 °C for 24 h. The pink colonies were further inoculated on Sorbitol MacConkey Agar (Hi-Media, Mumbai, India) and Eosin Methylene Blue agar (Hi-Media, Mumbai, India) and incubated at 37 °C for 24 h (Fig. S1, S2, S3). Colonies exhibiting a greenish metallic sheen on EMB agar and pale or pink colonies on SMAC agar were further characterized morphologically. Pale colonies on SMAC agar were identified only presumptively as O157: H7 candidates, which were later confirmed through serotyping. Both pale and pink colonies were selected for further analysis. Various biochemical and sugar fermentation tests—such as catalase, oxidase, indole, methyl red, Voges-Proskauer, citrate utilization, urease, nitrate reduction, triple sugar iron agar, and mannitol fermentation—were performed for further confirmation of *E. coli* isolates. Pure colonies were transferred to nutrient agar slants and stored at 4 °C for future use.

Quality control for isolation involved the use of both negative and positive controls. In negative controls sterile buffered peptone water, MacConkey agar, SMAC agar, and EMB agar were cultured in parallel with every batch to verify sterility. While, in positive controls reference *E. coli* strain (ATCC 25922) was cultured monthly to verify media performance. Media were replaced every 3 months to ensure viability.

### 2.3 Molecular characterization of *Escherichia coli* isolates

#### 2.3.1 DNA extraction

Bacterial DNA was extracted using the snap-chilled method (Sekhar et al., 2017). Each *E. coli* isolate was inoculated in 5 mL of Luria Bertani (LB) broth and incubated at 37 °C overnight under constant shaking. After incubation, 1 mL of culture was pelleted at 8000 rpm for 10 min at 4 °C. The pellet was washed three times with sterile normal saline and resuspended in 300 μL of sterile nuclease-free water. The suspension was boiled for (5–10) min in a water bath, followed by immediate chilling at −20 °C for 10 min. The lysate was centrifuged at 5000 rpm for 5 min, and the supernatant was used as template DNA for PCR. The purity and concentration of DNA were checked using a Nanodrop and gel electrophoresis, and DNA was stored at −20 °C.

#### 2.3.2 Molecular confirmation of *Escherichia coli* species by PCR targeting *yaiO* gene

All the isolates of *E. coli*, identified by both cultural and biochemical characterization were further subjected to PCR targeting *yaiO* gene for confirmation of *E. coli* species. The PCR cycling conditions for the detection of *E. coli* is listed in Table 1 (Ahmadi and Keshavarzi, 2018). Agarose gel electrophoresis (1.5% agarose in 1X TAE buffer) was used to evaluate the amplified PCR products. It was run at 80 V/cm for 45 min and stained with (0.5 μg/mL) ethidium bromide. Each sample’s PCR product (5–10 μL) were loaded in individual wells of gel alongside a positive control. For each run, the amplicon size was determined by using a 100 bp DNA ladder as the molecular weight marker. The products were visualized under a UV transilluminator and documented using a gel documentation system (AlphaImager).

**Table 1:** PCR condition for detection of *yaiO* gene of *E. coli.*

Target gene	Primer sequence	PCR product size	PCR conditions	PCR reaction volume (25 μL)
*yaiO*	F: TGATTTCCGTGCGTCTGAATG R: ATGCTGCCGTAGCGTGTTTC	115 bp	1. First cycle (95 °C for 3 min) 2. Subsequent 35 cycles of Denaturation (95 °C for 30 s), Annealing (58° C for 30s), & Elongation (72 °C for 45 s) 3. Finalextension(72 °C for 10 min)	12.5 μl of PCR master mix (1X, Thermo fisher) 10 picomole/μl of each primers forward and reverse 4.5 μL DNA template

#### 2.3.3 Multiplex PCR assay for detection of STEC and EPEC isolates

All *E. coli* isolates were screened by multiplex PCR for virulence genes (*stx1, stx2, eaeA*, and *hlyA*) as per Paton and Paton (1998) with suitable modifications. PCR was performed in 25 μL reaction mixtures in 200 μL thin-walled PCR tubes. Cycling conditions included: initial denaturation at 94 °C for 5 min, 30 cycles of denaturation (94 °C, 45 s), annealing (65 °C, 45 s), elongation (72 °C, 45 s), and a final extension at 72 °C for 5 min (Table 2). Duplicate PCR assays were run for every sample.

**Table 2:** PCR conditions for detection of STEC and EPEC isolates.

Target gene	Primer sequence	PCR product size	PCR conditions	PCR reaction volume (25 μL)
*stx_1_*	F-ATAAATCGCCATTCGTTGACTAC R- AGAACGCCCACTGAGATCATC	180	Step 1: 1st cycle(94 °C for 5 min) Step 2: Initial denaturation(94 °C for 45 s), subsequently 30 cycles: Annealing (65 °C for 45 s), elongation (72 °C for 45 s) Step 3: Final extension (72 °C for 5 min)	12.5 μL of PCR master mix (1X, Thermo fisher) 10 picomole/μl of each primers forward and reverse 4.5 μL DNA template
*stx_2_*	F- GGCACTGTCTGAAACTGCTCC R-TCGCCAGTTATCTGACATTCTG	255		
*eaeA*	F- GACCCGGCACAAGCATAAGC R- CCACCTGCAGCAACAAGAGG	384		
*hlyA*	F- GCATCATCAAGCGTACGTTCC R-AATGAGCCAAGCTGGTTAAGCT	534		

The amplified PCR products were analyzed by agarose gel electrophoresis (1.5% agarose in 1X TAE buffer) at 80 V/cm for 45 min and stained with ethidium bromide (0.5 μg/mL). 5–10 μL of the PCR product of each sample was loaded into individual wells alongside a positive control. A 100 bp DNA ladder served as the molecular weight marker for each run to determine amplicon size. The products were visualized under a UV transilluminator and documented by a gel documentation system (AlphaImager).

The quality control of PCR for detection of virulence genes involved positive and negative control. The reference *E.coli* O157: H7 strain (ATCC 35150) containing all the genes under study was used as a positive control in every PCR run and nuclease-free water without DNA template constituted a negative control.

### 2.4 Serogrouping of *Escherichia coli* isolates

All *E. coli* isolates that possessed at least one virulence gene were serotyped based on their somatic “O” antigen at the National Salmonella and *Escherichia* Centre, Central Research Institute, Kasauli, Himachal Pradesh, India.

### 2.5 Phenotypic characterization of STEC by Vero cell cytotoxicity assay

Phenotypic confirmation of STEC isolates was performed by assessing their cytotoxicity on Vero cell lines, following the protocols of Xia et al. (2010) and To and Bhunia (2019) with suitable modifications. The Vero cell line was maintained in Dulbecco’s Modified Eagle Medium (DMEM) supplemented with 10% fetal bovine serum and incubated at 37 °C with 5% CO₂. Confluent monolayers were obtained within 24 h.

For toxin preparation, STEC isolates were cultured in Luria–Bertani (LB) broth with and without ciprofloxacin (100 μg/mL) and incubated overnight at 37 °C. The supernatant was filtered (0.22 μm) and serially diluted (1:5 to 1:80) in DMEM. Each dilution (100 μL) was inoculated into wells containing Vero cell monolayers and cytopathic effects (CPEs) were examined under an inverted microscope at 12, 24, and 72 h post-infection. Negative control wells contained uninoculated cells and the reference strain used in this study served as positive control. After incubation, cells were fixed in 2% formalin, stained with 0.13% crystal violet, and washed with distilled water. The stained cells were dissolved in 33% acetic acid, and optical density (OD) was measured at 600 nm using an ELISA reader (BioTek Instruments). The cytotoxic dilution resulting in 50% cell death (CD₅₀) was estimated by comparing OD values with uninoculated control wells through linear regression of OD with dilution rate. The reference *E. coli* O157: H7 (ATCC 35150) producing active Shiga toxin was used a positive control for every run and uninoculated Vero cells in DMEM medium was used as a negative control. Assays were performed in triplicate and a standard cut-off value of OD < 0.45 was defined as cell-death threshold.

### 2.6 Antibiotic sensitivity assay

All STEC and EPEC isolates were tested for antibiotic susceptibility using the disc diffusion method (Bauer et al., 1966) against 19 antibiotics commonly used in human and veterinary medicine (Supplementary Table S2), following CLSI guidelines (CLSI Supplement M100, 2020).

Each isolate was grown overnight in LB broth at 37 °C. The turbidity of the bacterial broth was adjusted to a 0.5 McFarland standard, corresponding to an approximate bacterial concentration of 1.5 × 10^8^ CFU/mL. A 100 μL aliquot of the overnight broth culture was evenly spread over Mueller-Hinton (MH) agar plates using a sterile spreader. Antibiotic discs were placed on the inoculated MHA surface at approximately 2 cm intervals using sterile forceps. The plates were then incubated at 37 °C overnight, after which the diameters of the zones of inhibition were measured. The results were compared with the zone size interpretative chart provided by the manufacturer, and the isolates were classified as sensitive, intermediate, or resistant based on CLSI charts. The *E. coli* ATCC 25922 was used as the standard quality control strain and the testing was done in triplicate for each isolate. The antibiotic discs were stored at 2–8 °C and checked for expiry dates before use.

### 2.7 Detection of antibiotic resistance genes in STEC and EPEC isolates

All STEC and EPEC isolates showing complete resistance to tetracycline, sulfonamide, and ampicillin were screened for resistance genes (*tetA, tetB, sul1,* and *CITM*) using multiplex PCR as per Ranjbar et al. (2018), with suitable modifications.

PCR was performed in 200 μL thin-walled tubes in a 25 μL reaction volume using a thermal cycler. Cycling conditions included an initial denaturation at 94 °C for 8 min, followed by 32 cycles of denaturation (95 °C, 60 s), annealing (55 °C, 70 s), and elongation (72 °C, 2 min), with a final extension at 72 °C for 8 min (Table 3). Amplified PCR products were analyzed by electrophoresis on 1.5% agarose gels in 1 × TAE buffer at 80 V/cm for 45 min, stained with ethidium bromide (0.5 μg/mL), and visualized under a UV transilluminator. Ten microliters (10 μL) of each PCR product were loaded per well alongside a 100 bp DNA ladder for size determination, and images were captured using a gel documentation system (AlphaImager). Positive and negative controls were included in each run and duplicate PCR assays were run for every sample.

**Table 3:** PCR conditions for detection of antibiotic resistance genes in STEC and EPEC isolates.

Target gene	Primer sequence	PCR product size	PCR conditions	PCR reaction volume (25 μL)
*tetA*	F: GGTTCACTCGAACGACGTCA R: CTGTCCGACAAGTTGCATGA	577	Step 1:1^st^ cycle (94 °C for 8 min). Step 2: Subsequent 32 cycles: Denaturation (95 °C for 60s), Annealing (55 °C for 70s), Elongation (72 °C for 2 min). Step 3 Final extension: (72 °C for 8 min)	12.5 μL of PCR master mix (1X, Thermo fisher) 10 mM of each primers forward and reverse 4.5 μl DNA template
*tetB*	F: CCTCAGCTTCTCAACGCGTG R: GCACCTTGCTGATGACTCTT	634		
*Sul*1	F: TTCGGCATTCTGAATCTCAC R: ATGATCTAACCCTCGGTCTC	822		
*CITM*	F: TGGCCAGAACTGACAGGCAAA R: TTTCTCCTGAACGTGGCTGGC	462		

### 2.8 Statistical analysis

This was a descriptive, cross-sectional prevalence study without hypothesis testing. Data were entered in a Microsoft Excel spread sheet and subjected to a distribution test (Chi-square test). Statistical analyses were performed using SPSS/20.0 software (SPSS Inc., Chicago, IL). Chi-square tests assessed the mean differences in prevalence rates, ANOVA compared mean cytotoxic effects, and in case of low counts Fisher’s exact test was applied. Differences in *E. coli* prevalence and virulence gene frequencies among milk and milk products were considered statistically significant at *p* < 0.05.

## 3 Results

### 3.1 Prevalence of *E. coli* in raw milk and milk product samples

Among the 260 raw milk samples, 81 were positive for *E. coli*, and among the 420 milk product samples, 115 were positive (Table 4). The overall combined prevalence rate of *E. coli* in milk and milk products was 28.82%. The prevalence rate in raw milk was 31.15%, whereas in milk products, it was 27.38% (95% CI: 23.3–31.7%). The lowest prevalence of *E. coli* was observed in ghee (10%), while the highest was in dahi (43.0%). The prevalence rates were significantly different among all milk and milk product types (χ^2^ = 25.34, *p* < 0.001). However, the difference between milk and lassi was not statistically significant (31.15% vs. 32.0%, *p* = 0.87).

**Table 4:** Prevalence of *E. coli* in raw milk and milk products.

S. No.	Sample type	Number of samples	No. of *E. coli* isolated	Percentage prevalence (%)	95% Confidence interval
1	Raw milk	260	81	31.15	25.4–37.2
2	Ghee	100	10	10.0	4.8–17.8
3	Paneer	120	30	25.0	17.2–34.3
4	Lassi	100	32	32.0	22.8–42.2
5	Dahi	100	43	43.0	33.0–53.2
	Total	680	196	28.82	25.5–32.2%

### 3.2 Detection of virulence genes (*stx1, stx2, eaeA, hlyA*) in STEC and EPEC isolates

**Figure 1:**
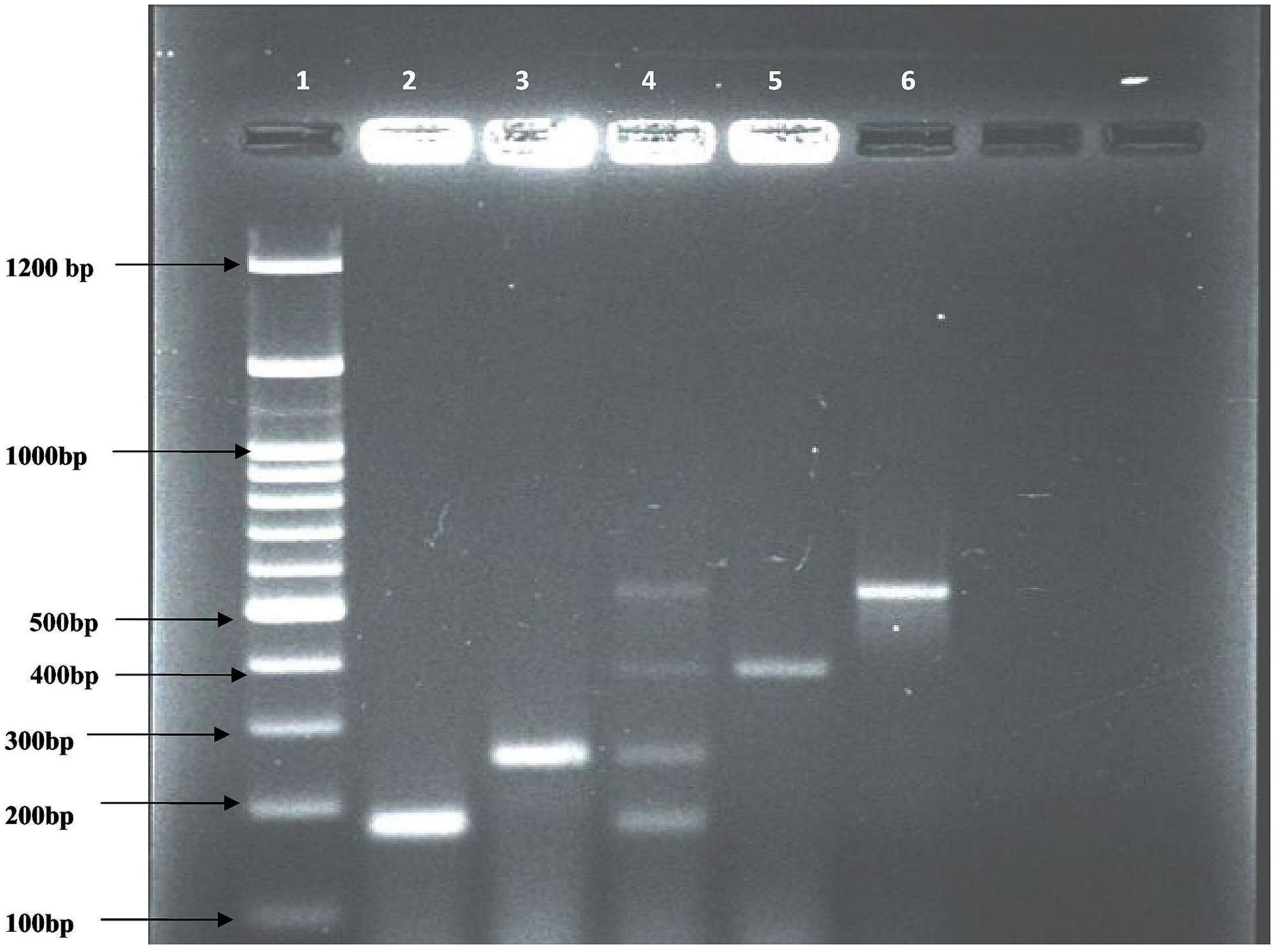
Multiplex PCR results for virulence genes. Lane 1 = 100 bp DNA ladder; lane 2 = positive isolate for *stx1* gene (180 bp); lane 3 = positive isolate for *stx2* gene (255 bp); lane 4 = positive control for all the 4 genes: *stx1, stx2, eaeA and hlyA*; *l*ane 5 = positive isolate for *eaeA* gene (384 bp); lane 6 = positive isolate for *hlyA* gene (534 bp).

All 196 pure *E. coli* isolates were screened by multiplex PCR for the detection of four virulence genes – *stx1, stx2, eaeA,* and *hlyA* (Figure 1) and 78 (39.8, 95% CI: 32.9–47.0%) harbored at least one virulence gene. Out of the 81 isolates of *E. coli* recovered from milk samples, 45 (55.56, 95% CI: 44.5–66.1%) were positive for at least one virulence gene, while out of the 115 isolates from milk product samples, 33 (28.70, 95% CI: 20.7–37.9%) were positive (Supplementary Tables S3, S4). The differences were statistically significant (χ^2^ = 15.78, *p* < 0.001). Among the 45 positive isolates from milk, 7 (15.56%) were identified as EPEC, and 38 (84.40%) as STEC. Similarly, among the 33 positive isolates from milk products, 5 (15.15%) were EPEC, and 28 (84.85%) were STEC. The overall prevalence of STEC and EPEC pathotypes among 78 virulence-positive isolates is 84.6 and 15.4%, respectively.

The prevalence of STEC/EPEC in milk, based on total samples collected, was 17.30%. For milk products, the prevalence was 3.0% for ghee, 6.67% for paneer, 10.0% for lassi, and 12.0% for dahi, with an overall prevalence of 7.86%. Based on total samples collected, the combined prevalence of STEC/EPEC in milk and milk products was 11.47%. Among the detected virulence genes, *stx1* was the most predominant in both raw milk and milk products, while *hlyA* was the least abundant (Tables 5, 6). Overall, the prevalence of virulence genes among STEC and EPEC isolates was highest for *stx1* (64.1%), followed by *eaeA* (43.6%), *stx2* (41.0%), and *hlyA* (33.3%). The overall frequency distribution of virulence genes was significantly different (χ^2^ = 18.42, *p* < 0.001). However, no significant difference between *stx2* and *eaeA* was observed (*p* = 0.78).

**Table 5:** Prevalence pattern of different virulence genes in the STEC and EPEC isolates.

Sl. No.	Type of sample	STEC/EPEC isolates	Virulence gene	Virulence gene frequency	Percentage based on STEC/EPEC isolates
1.	Raw milk	45	*stx1*	24	53.3
			*stx2*	19	42.2
			*HlyA*	18	40.0
			*eaeA*	22	48.9
2.	Ghee	03	*stx1*	03	100.0
			*stx2*	02	66.7
			*HlyA*	01	33.3
			*eaeA*	01	33.3
3.	Paneer	08	*stx1*	06	75.0
			*stx2*	03	37.5
			*HlyA*	01	12.5
			*eaeA*	04	50.0
4.	Lassi	10	*stx1*	08	80.0
			*stx2*	04	40.0
			*HlyA*	02	20.0
			*eaeA*	04	40.0
5	Dahi	12	*stx1*	09	75.0
			*stx2*	04	33.3
			*HlyA*	04	33.3
			*eaeA*	03	25.0
	Total	78			

**Table 6:** Overall prevalence pattern of different virulence genes in the STEC and EPEC isolates (*n* = 78).

Virulence gene	Virulence gene frequency	Percentage based on STEC/EPEC isolates
*stx1*	50	64.1
*stx2*	32	41.0
*hyla*	26	33.3
*eaeA*	34	43.6

### 3.3 Serotyping of STEC isolates

Of the 78 *E. coli* isolates (45 from milk and 33 from milk products) positive for at least one virulence gene, 22 distinct O-serogroups were identified. The most prevalent serogroups were O18 (*n* = 18), followed by O126 (*n* = 7), O17 (*n* = 7), O120 (*n* = 7), and O111 (*n* = 7). Other serogroups included O26, O134, O119, O5, O20, O135, O63, O157, O101, O64, O88, O76, O84, O11, O86, and O121, with six isolates being untypeable (Supplementary Tables S5, S6).

Among milk isolates, O18, O120, and O17 predominated, while milk product isolates were largely O18, O111, and O126. Notably, serotypes O18, O120, O63, O134, O64, O88, O76, and O84 have rarely been reported in milk or dairy products.

### 3.4 Phenotypic characterization by Vero cell cytotoxicity

All 66 STEC isolates positive for stx1, stx2, or both, produced visible cytopathic effects on Vero cell monolayers. Morphological progression involved rounding of cells after 12 h, followed by syncytia formation and multinucleated giant cells at 24 h, and complete cell detachment and lysis at 72 h (Fig S5a-S5j). The degree of cytotoxicity was dose- and incubation time-dependent. The 50% cytotoxic dilution (CD₅₀) was consistently observed at a 1:40 dilution (Supplementary Table S7). The cytotoxicity got enhanced with supernatants from ciprofloxacin-treated STEC cultures compared to untreated cultures. Hence, ciprofloxacin-induced preparations yielded lower CD₅₀ values, indicating enhanced toxin expression.

### 3.5 Antibiotic sensitivity test

All 78 isolates (45 from milk and 33 from milk products) of STEC and EPEC were subjected to antibiotic sensitivity testing against 19 different antibiotics commonly used in clinical settings in humans and animals. The results were recorded as sensitive, intermediate, and resistant after measuring the zones of inhibition and comparing the recorded values with the CLSI guideline values. The overall resistance profile is depicted in Table 7. The STEC and EPEC isolates recovered from this study showed significantly (*p* < 0.05) low resistance to imipenem (1.3%), gentamicin (6.4%), and nalidixic acid (7.7%), whereas higher resistance was observed against ampicillin (88.5%), tetracycline (88.5%), oxytetracycline (87.2%), cephalothin (87.2%), and sulphadiazine (80.8%).

**Table 7:** Antimicrobial resistance pattern of STEC and EPEC isolates from milk and milk products (*n* = 78).

Sl. No.	Antimicrobial agent	Concentration per disc in μg	Sensitive	Intermediate	Resistant	% Resistant	95% Confidence interval
1	Ampicillin	10	8	1	69	88.5	79.3–94.8
2	Tetracycline	10	7	2	69	88.5	79.3–94.8
3	Oxytetracycline	30	6	9	68	87.2	77.8–93.8
4	Cephalothin	30	6	4	68	87.2	77.8–93.8
5	Sulphadiazine	100	9	6	63	80.8	70.3–88.9
6	Enrofloxacin	10	21	8	49	62.8	51.3–73.3
7	Vancomycin	10	22	22	34	43.6	32.3–55.3
8	Erythromycin	30	16	29	33	42.3	31.3–53.8
9	Amoxicillin	30	25	21	32	41.0	30.2–51.9
10	Chloramphenicol	30	33	14	31	39.7	28.9–51.3
11	Azithromycin	15	24	24	30	38.5	27.8–50.0
12	Streptomycin	10	21	30	27	34.6	24.3–46.2
13	Ceftriaxone	30	35	17	26	33.3	23.1–44.8
14	Co-trimoxazole	10	27	25	26	33.3	23.1–44.8
15	Ceftazidime	30	36	19	23	29.5	19.8–40.8
16	Cefixime	5	29	27	22	28.2	18.8–39.2
17	Nalidixic acid	30	69	4	6	7.7	3.0–15.3
18	Gentamicin	120	67	6	5	6.4	2.1–14.2
19	Imipenem	10	71	6	1	1.3	0.0–6.8

Different degrees of sensitivity based on the zones of inhibition were observed among the STEC and EPEC isolates from milk samples against different antibiotics. The maximum number of isolates (40; 88.9%) were sensitive to imipenem, with just five isolates showing intermediate sensitivity, and no resistant isolates were observed against imipenem. The sensitivity pattern of isolates against imipenem was followed by nalidixic acid (39; 86.7%), gentamicin (36; 80.0%), and so on. The least effective antibiotic was ampicillin, with 39 isolates (86.7%) showing complete resistance, one isolate showing intermediate sensitivity, and only five isolates showing sensitivity. The resistance pattern to ampicillin was closely followed by sulphadiazine (38; 84.4%), cephalothin (38; 84.4%), tetracycline (36; 80.0%), oxytetracycline (35; 77.8%), enrofloxacin (31; 68.9%), and so on (Supplementary Table S8; Supplementary Figure S4).

In the ghee samples, all the STEC isolates were resistant (100%) to oxytetracycline, sulphadiazine, tetracycline, ampicillin, and cephalothin, whereas all the isolates were sensitive to co-trimoxazole, gentamicin, imipenem, nalidixic acid, and erythromycin (Supplementary Table S9). In the paneer samples, all the STEC and EPEC isolates were resistant (100%) to oxytetracycline, tetracycline, and cephalothin. The resistance pattern was followed by isolates in decreasing order against vancomycin/ampicillin (87.5%), amoxicillin (75.0%), enrofloxacin/streptomycin/ceftriaxone (62.5%), and so on. The most effective antibiotics, with 100% susceptibility of isolates, were gentamicin, imipenem, and nalidixic acid (Supplementary Table S10).

In the lassi samples, all the STEC and EPEC isolates were resistant to oxytetracycline, tetracycline, and ampicillin. The resistance pattern in decreasing order was observed against sulphadiazine (80%), amoxicillin/vancomycin/co-trimoxazole/azithromycin/cephalothin (70%), and so on. The most effective antibiotics, to which all isolates were sensitive, were gentamicin and nalidixic acid, followed by imipenem, against which 90% of isolates were sensitive (Supplementary Table S11).

In dahi samples, all the STEC and EPEC isolates were resistant to oxytetracycline, sulphadiazine, tetracycline, and cephalothin. The resistance pattern in decreasing order was observed against azithromycin (91.7%), ampicillin (83.3%), streptomycin/erythromycin (75%), and so on. The most effective antibiotics, showing maximum inhibition of isolates, were imipenem (91.7%), gentamicin (83.3%), and nalidixic acid (75%) (Supplementary Table S12).

### 3.6 Antibiotic resistance gene detection in STEC and EPEC isolates

**Figure 2:**
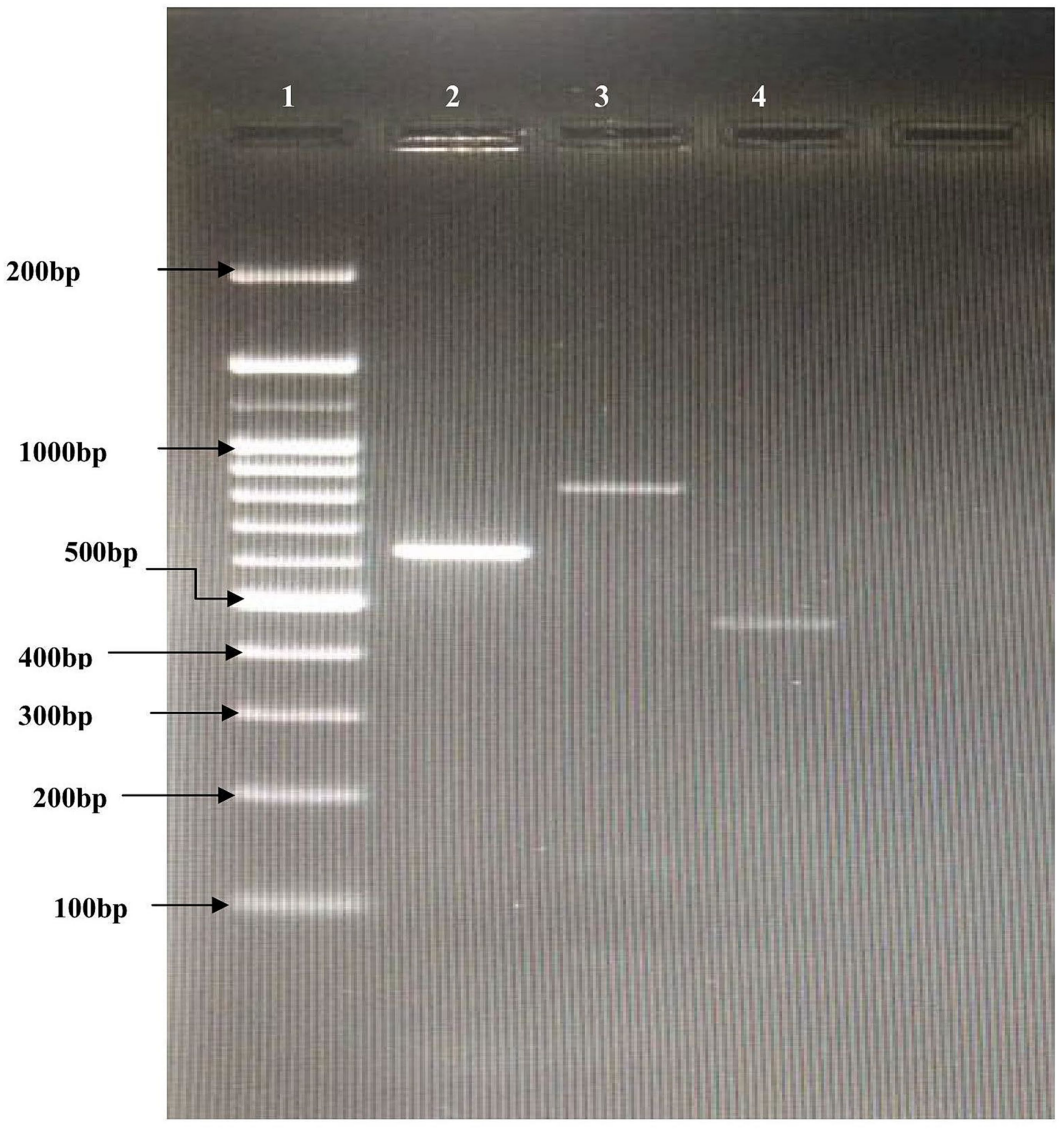
Multiplex PCR results for antibiotic resistance genes. Lane 1 = 100 bp DNA ladder; lane 2 = *tetB*, *l*ane 3 = *sul1*; *l*ane 4 = *CITM.*

Among the 78 STEC and EPEC isolates, 40 (51.3%) were phenotypically resistant to all three antibiotics – tetracycline, sulphadiazine, and ampicillin. Out of these 40 isolates, 38 (95%) were positive for at least one resistance gene - *tetB, sul1*, and *CITM*. All 38 isolates (100%) tested positive for the *tetB* resistance gene, while the *sul1* and *CITM* resistance genes were detected in 8 isolates (20%) and 10 isolates (25%), respectively. None of the isolates were positive for the *tetA* gene (Figure 2).

## 4 Discussion

Shiga-toxigenic *Escherichia coli* (STEC) strains are among the most important bacteria that cause foodborne diseases worldwide, resulting in a high number of hospitalizations (Ranjbar et al., 2018; Schallan et al., 2011; Rafee et al., 2025). Milk and milk products constitute an integral part of human diets and are major sources of *E. coli* infections in humans. In the present study, 31.15% of raw milk samples and 27.38% of milk product samples were contaminated with *E. coli* strains. The highest *E. coli* contamination was observed in dahi (43%) and the lowest in ghee (10%). The presence of *E. coli* in milk and milk products is an important indicator of fecal contamination and unhygienic practices. In line with the results of the present study, the prevalence of different strains of *E. coli* in milk and milk products ranged from 21 to 77% in a study from Egypt (Ombarak et al., 2016). The overall prevalence in this study was slightly lower than that reported in milk and milk products in Iran (30.16%) (Ranjbar et al., 2018) and much lower than that reported from Zimbabwe (66.6%) (Gran et al., 2003) and Pakistan (50–65%) (Soomro et al., 2002). The prevalence in this study was marginally higher than other reports from Iran (26%) (Farzan et al., 2012) and South Africa (23.3%) (Lues et al., 2003). Among the milk products in this study, the higher prevalence in dahi may be due to the use of unpasteurized milk for its production, which facilitates bacterial survival compared to ghee. In ghee, the 10% prevalence can be attributed to post-processing contamination, as ghee production involves exposure to high temperatures for extended periods, which reduces bacterial survival.

*E. coli* is generally a normal inhabitant of the large intestine in humans and animals, and fecal contamination is the primary source in raw milk and milk products during milking under poor hygienic conditions. The presence of non-pathogenic *E. coli* in dairy products is not a major concern for consumers. However, over time, *E. coli* acquires virulence genes, rendering it pathogenic and capable of causing a variety of diseases in both animals and humans (Begum et al., 2018). Several virulence factors have been characterized in STEC/EPEC, with the most commonly encountered being Shiga toxins (*stx1* and *stx2*), intimin (*eaeA*), and hemolysin (*hlyA*). Shiga toxins and hemolysin induce lesions, while intimin facilitates attaching and effacing lesions on epithelial surfaces.

In the present study, the overall prevalence of *E. coli* positive for at least one virulence gene was 39.8%. The raw milk samples showed higher prevalence (55.56%) compared to the milk product isolates(28.7%). The prevalence of EPEC isolates in milk and milk products was 15.56 and 15.15%, respectively, with the remainder being STEC isolates. The virulence gene *stx1* was the most predominant in milk and milk products, while *hlyA* was the least abundant among the STEC and EPEC isolates. The prevalence of STEC has been reported to be higher in developed countries than in developing countries (Schallan et al., 2011), possibly due to underreporting or limited research in developing regions. Although the overall prevalence of *E. coli* in milk and milk products in this study (28.82%) was slightly lower than that in Iran (30.16%) (Ranjbar et al., 2018), the prevalence of STEC/EPEC was higher (39.8% vs. 35.35%). Similarly, in Sikkim and Arunachal Pradesh, 16.21% EPEC and 83.79% STEC were recovered from yak milk samples (Dutta et al., 2011). The higher prevalence of STEC in India may be attributed to unhygienic milk production practices, inadequate supply chain management, the widespread use of unpasteurized milk for direct sale and milk product preparation, and the environmental acquisition of virulence genes by *E. coli* (Purwar et al., 2016). Compared to milk products, raw milk in this study revealed a higher prevalence of STEC (55.55%), consistent with reports from Iran (50%) (Ranjbar et al., 2018) and Egypt (76.4%) (Ombarak et al., 2016).

In serotyping of *Escherichia coli*, the isolates carrying virulence genes were found to belong to 22 O-serogroups, with O18 most prevalent, followed by O126, O17, O120, and O111. Such broad diversity agrees with recent reports showing an increasing role of non-O157 *E. coli* (e.g., O26, O111, O121, O145) in foodborne illness associated milk and dairy products (Miko et al., 2014; Ranjbar et al., 2018). The dominance of O18, typically linked to extra-intestinal pathogenic *E. coli*, suggests regional adaptation or environmental persistence in local cattle population, warranting further investigation. Notably, serotypes O120, O63, O134, O76, and O84 have rarely been reported in milk or dairy products but cannot be declared as “emerging” without longitudinal study from the same region involving broader molecular surveillance integrating serogrouping, virulence, and resistance profiling. They shall be considered “detected” in the present study rather than definitively “emerging.” Similar regional variability in non-O157 STEC distribution has been reported from India, where recent studies document diverse non-O157 serogroups in raw milk and cattle feces and link serogroup patterns to hygiene, seasonality and antimicrobial pressure (Joseph and Kalyanikutty, 2022; Bist et al., 2023). Serogroup heterogeneity is epidemiologically important because genetic exchange among strains can generate novel virulent or resistant lineages (Henrot and Petit, 2022). Broader molecular surveillance integrating serogrouping, virulence, and resistance profiling is therefore essential to monitor evolving *E. coli* populations in dairy environments.

In vero-cell cytotoxicity assay, all *stx*-positive isolates caused clear cytopathic effects on Vero cells – cell rounding, syncytia formation, and lysis – confirming expression of biologically active Shiga toxin. The time- and dose-dependent damage observed agrees with previous findings (To and Bhunia, 2019; Xia et al., 2010). Toxin production was potentially enhanced by ciprofloxacin induction, consistent with evidence that DNA-damaging antibiotics activate prophages carrying *stx* genes and increase toxin release (Henrot et al., 2022; Bloch et al., 2024). These results underline the need for cautious antibiotic use in livestock to avoid stimulating toxin production. Combining molecular detection with functional assays provides stronger confirmation of pathogenic potential. Demonstration of active toxin production in milk-derived isolates highlights the zoonotic significance of STEC/EPEC in unpasteurized dairy products and emphasizes strict hygienic practices and regular microbiological monitoring (Joseph and Kalyanikutty, 2022). However, it is noteworthy that quantitative assessment of toxin concentration by ELISA or qPCR was not carried out in this study. The results were based on qualitative morphological observations and semi-quantitative OD measurements.

Another important aspect of *E. coli* infections in humans and animals is the emergence of antibiotic resistance. The rise of antibiotic resistance in STEC has been attributed to several factors, with the indiscriminate use of antibiotics in animals for therapy and growth promotion being a major contributor. In the present study, STEC and EPEC isolates were least resistant to imipenem (1.3%), gentamicin (6.4%), and nalidixic acid (7.7%), while showing maximum resistance to ampicillin (88.5%), tetracycline (88.5%), oxytetracycline (87.2%), cephalothin (87.2%), and sulphadiazine (80.8%). Numerous studies have reported antibiotic resistance in STEC and EPEC against commonly used antibiotics (Elmonir et al., 2021; Ismail and Abutarbush, 2020; Sultana et al., 2021; Adzitey et al., 2022; Elafify et al., 2022; Islam et al., 2016). However, consistency in resistance levels is lacking, likely due to variations in antibiotic types tested and differing regional regulations on antibiotic use. Notably, STEC/EPEC isolates in this study were resistant to antibiotics not commonly used in veterinary medicine in India, indicating environmental transfer of resistance elements from humans to animals.

Resistance elements are acquired by STEC or EPEC either through mutations under antibiotic selection pressure or via horizontal gene transfer from the environment. In the present study, 95% (*n* = 38) of STEC/EPEC isolates resistant to tetracycline, sulphadiazine, and ampicillin harbored *tetB, sul1*, and *CITM* resistance genes. Strong correlation between phenotypic and genotypic resistance against tetracycline was observed in this study. All 38 genotype-positive isolates carried *tetB*, but *tetA* was completely absent, suggesting exclusive *tetB*-mediated tetracycline resistance in the isolates. However, weak to moderate correlation was observed in case of sulfonamide and ampicillin resistance. Only 20% of phenotypically sulfonamide-resistant isolates carried *sul1* and 25% of phenotypically ampicillin-resistant carried *CITM*, which indicates the dominance of other un-investigated resistances mechanisms. The authors acknowledge that the limited gene panel in this study does not capture the full spectrum of resistance determinants. Similar findings have been reported elsewhere, where 79.8% of O157 and 91.7% of non-O157 STEC isolates carried at least one resistance gene, regardless of phenotype (Srinivasan et al., 2007). The increasing antimicrobial resistance in *E. coli* may be due to plasmid-mediated horizontal transfer of resistance genes within food chain in between the microbial populations (Kousar et al., 2025). However, no molecular fingerprinting, such as Whole Genome Sequencing, Pulsed-Field Gel Electrophoresis, or Multilocus Sequence Typing was performed; thus clonal linkage between virulent and MDR strains could not be inferred.

The extent of correlation between phenotypic and genotypic resistance in STEC and EPEC has varied widely across studies, likely due to differences in antibiotic usage patterns and environmental conditions influencing the acquisition of resistance elements in different geographical regions. In contrast to this study, all STEC isolates from milk in Bangladesh (Bag et al., 2021) and Egypt (Younis et al., 2021) were positive for the *tetA* gene, while none in this study harbored it. A study in Iran found 64.7% of STEC isolates positive for *tetA*+*tetB* genes in milk and dahi samples (Momtaz et al., 2012), suggesting that tetracycline resistance in this study was exclusively mediated by *tetB*. Additionally, 98% of *E. coli* isolates in Iran carried the CITM gene (Ranjbar et al., 2018), compared to 25% in this study, while 20% *sul1* positivity here exceeded the 9% reported in Egypt (Algammal et al., 2022).

An emerging aspect of antibiotic resistance in STEC and EPEC, highlighted in recent reports, is the potential association between virulence factors and antibiotic resistance; however, this relationship has yet to be definitively established and warrants further research.

## 5 Conclusion

This cross-sectional surveillance study highlights the significant contamination of milk and milk products in Uttarakhand, Northern India, with virulent and multidrug-resistant *Escherichia coli* strains, including STEC and EPEC. The predominance of the *stx1* gene among virulence factors, together with diverse non-O157 serogroups such as O18, O111, O120, and O126, underscores the emergence of region-specific pathogenic variants. Demonstrated cytotoxicity on Vero cells confirms the active expression of Shiga toxin, reinforcing their zoonotic and food safety relevance. The high resistance levels to ampicillin, tetracycline, cephalothin, and sulfonamide coupled with the widespread presence of the *tetB*, *sul1*, and *CITM* genes may reflect growing antimicrobial exposure patterns historically prevalent in the dairy ecosystem of the region. These findings emphasize the urgent need for strict hygienic milk handling, rational antibiotic use in livestock, and continuous molecular surveillance to mitigate public health risks associated with pathogenic *E. coli* in the dairy sector.

## Data Availability

The original contributions presented in the study are included in the article/Supplementary material, further inquiries can be directed to the corresponding author.
