# Supplementary material for: Molecular detection, serotyping, cytotoxicity, and antimicrobial resistance of STEC and EPEC isolated from milk and milk products in northern India
Source: Front Microbiol. 2026 Feb 18;17:1748367. doi: 10.3389/fmicb.2026.1748367 (PMC12957279; doi:10.3389/fmicb.2026.1748367)
Supplement: Supplementary file 1 [file Table_1.DOCX]

**Supplementary Tables and Figures**

| **Table S1. Sample collection from different locations in Uttarakhand** | | |
| --- | --- | --- |
| **S. No.** | **Sample type** | **Number of samples** |
|  | Raw milk | 260 |
|  | Ghee | 100 |
|  | Paneer | 120 |
|  | Lassi | 100 |
|  | Dahi | 100 |
|  | Total | 680 |

**
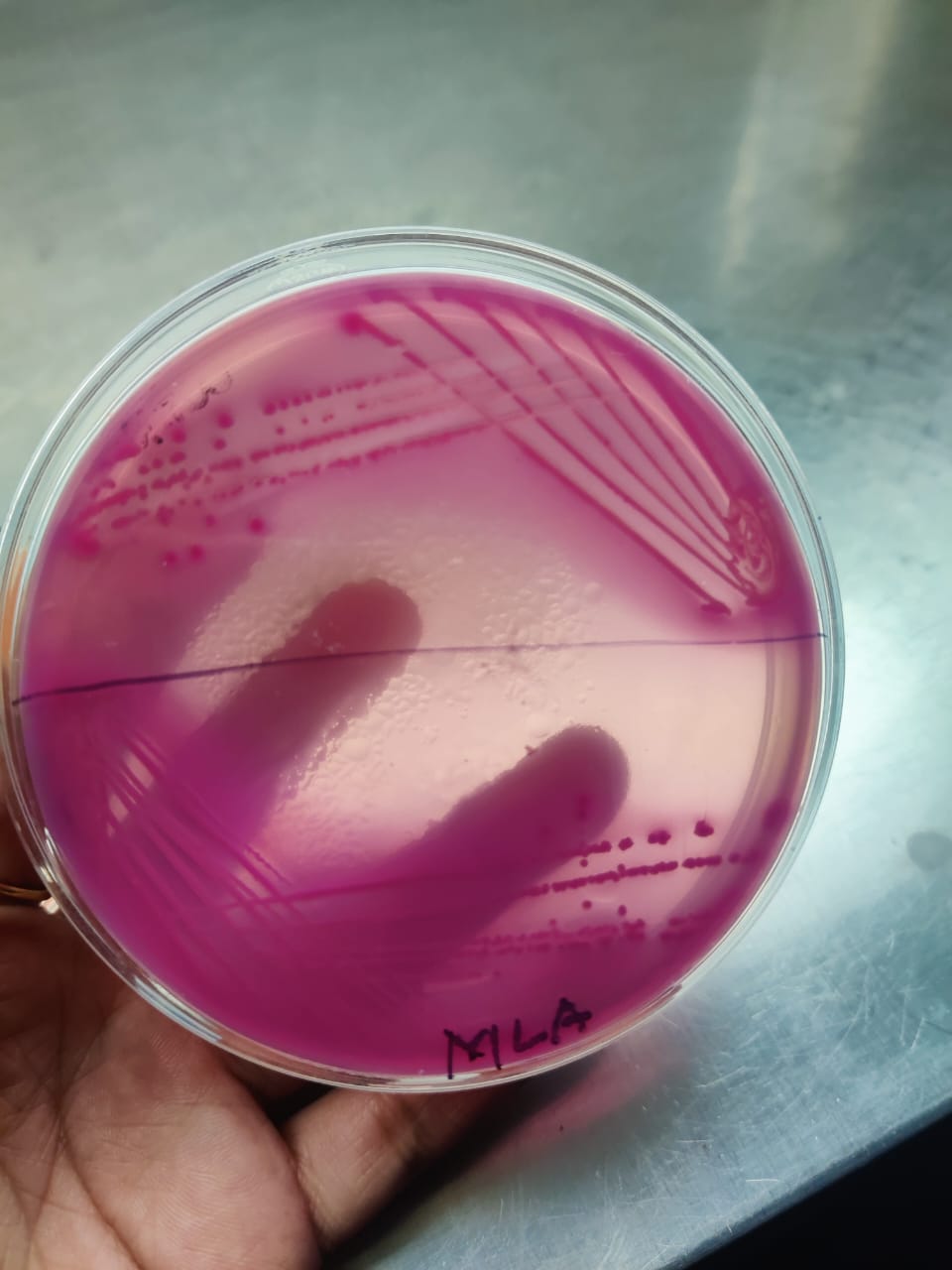

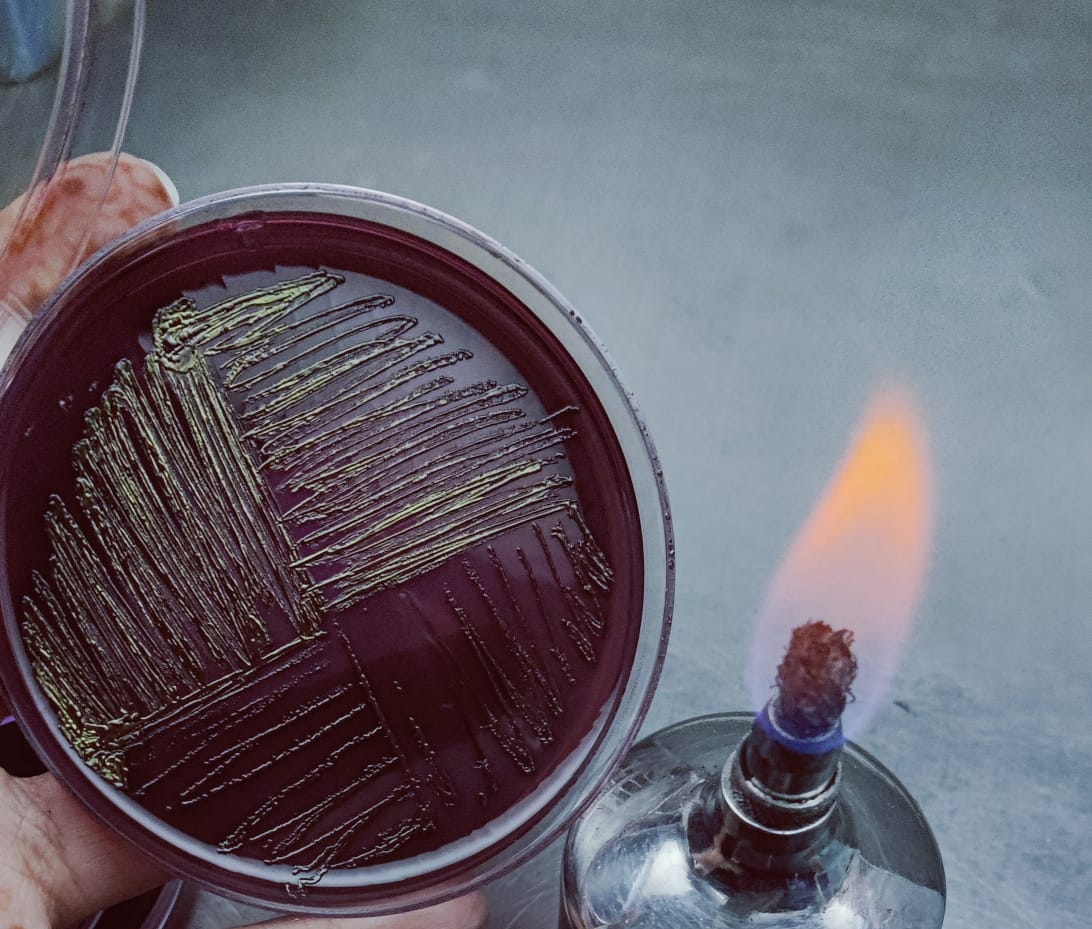
**

| **Fig S1: Lactose fermenting pink coloured colonies of *E. coli* on MacConkey agar Fig** |  | **Fig S2: Greenish metallic sheen colonies of *E. coli* on eosin methylene blue agar** |
| --- | --- | --- |

**
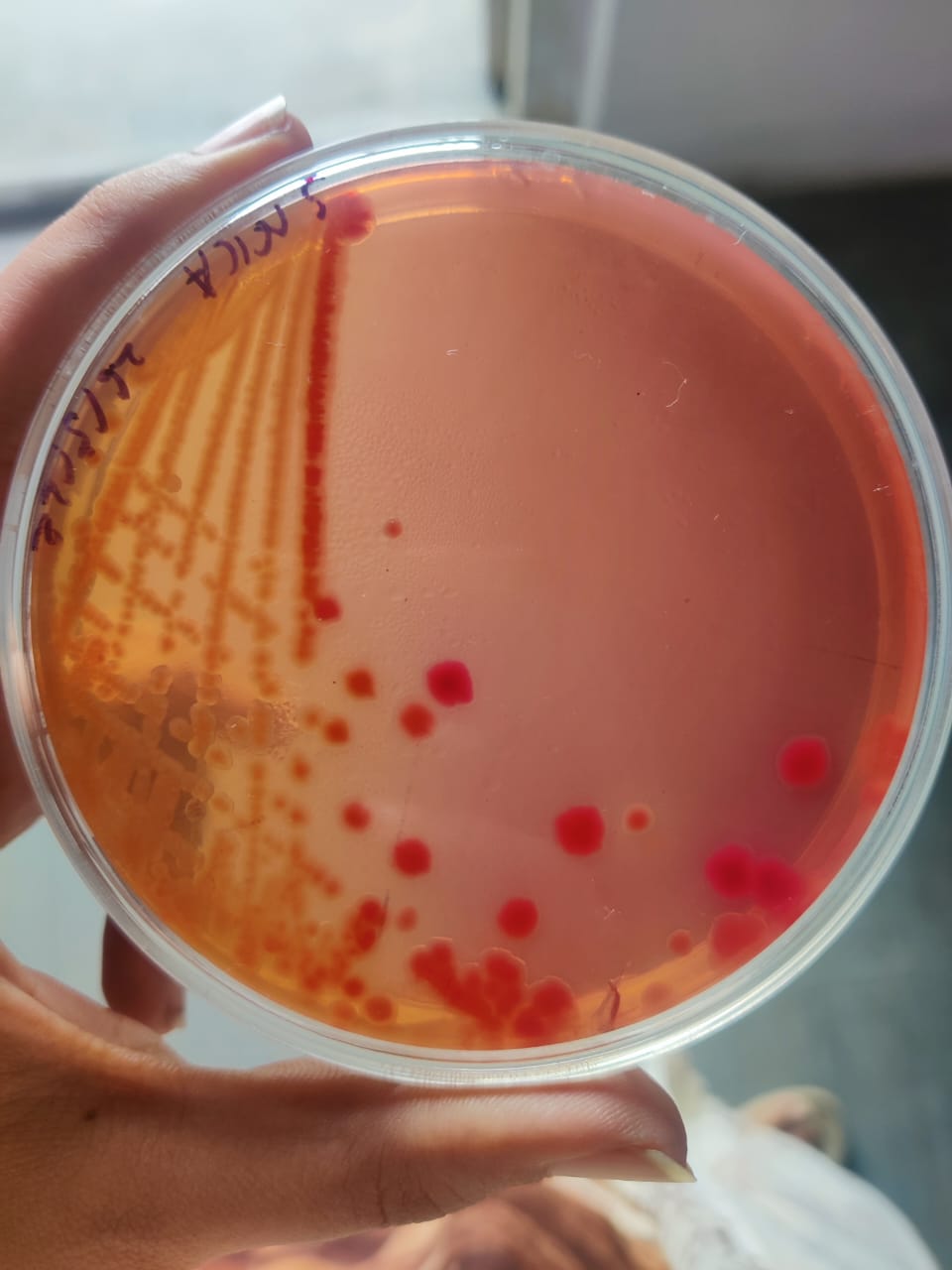
**

| **Fig S3: Pink and pale coloured colonies of *E. coli* non-O157 on SMAC agar Fig** | |  |  |  |  |
| --- | --- | --- | --- | --- | --- |
| **Table S2. Antibiotics used in sensitivity testing** | | | | | |
| **S. No.** | | **Antimicrobial reagent** | | | **Concentration per disc** |
| 1. | | Gentamicin(HLG) | | | 120 µg |
| 2. | | Ceftriaxone(CTR) | | | 30 µg |
| 3. | | Cefixime(CFM) | | | 5 µg |
| 4. | | Cephalothin(CEP) | | | 30 µg |
| 5. | | Ceftazidime(CAZ) | | | 30 µg |
| 6. | | Chloramphenicol(C) | | | 30 µg |
| 7. | | Imipenem(IPM) | | | 10 µg |
| 8. | | Enrofloxacin(EX) | | | 10 µg |
| 9. | | Clotrimazole(CC) | | | 10 µg |
| 10. | | Erythromycin(E) | | | 30 µg |
| 11. | | Tetracycline(TE) | | | 10 µg |
| 12. | | Nalidixic Acid(NA) | | | 30 µg |
| 13. | | Azithromycin(AZM) | | | 15 µg |
| 14. | | Sulphadiazine(SZ) | | | 100 µg |
| 15. | | Oxytetracycline(O) | | | 30 µg |
| 16. | | Amoxycillin(AMX) | | | 30 µg |
| 17. | | Ampicillin(AMP) | | | 10 µg |
| 18. | | Streptomycin(S) | | | 10 µg |
| 19. | | Vancomycin(VA) | | | 10 µg |

| **Table S3.Distribution of the virulence genes in *E. coli* isolated from the milk samples** | | | | | |
| --- | --- | --- | --- | --- | --- |
| **No. of milk samples collected** | **Number of *E. coli* isolated** | **No. of *E. coli* isolates positive for atleast one virulence gene** | **Virulence gene recovered** | **No. of positive isolates recovered from** | **Percentage prevalence** |
| 260 | 81 | 45 | *stx1* | 07 | 8.64 |
|  |  |  | *stx2* | 03 | 3.70 |
|  |  |  | *HlyA* | 04 | 4.94 |
|  |  |  | *eaeA* | 04 | 4.94 |
|  |  |  | *stx1+stx2* | 03 | 3.70 |
|  |  |  | *stx1+eaeA* | 06 | 7.41 |
|  |  |  | *Stx1+hlyA* | 01 | 1.23 |
|  |  |  | *stx1+eaeA+hlyA* | 01 | 1.23 |
|  |  |  | *stx2+eaeA* | 02 | 2.47 |
|  |  |  | *stx2+hlyA* | 05 | 6.17 |
|  |  |  | *eaeA+hlyA* | 03 | 3.70 |
|  |  |  | *stx1+stx2+eaeA* | 02 | 2.47 |
|  |  |  | *stx1+stx2+eaeA+hlyA* | 04 | 4.94 |
|  |  |  | **Total** | 45 | 55.56 |

| **Table S4. Distribution of virulence genes in *E. coli* isolated from the milk product samples** | | | | | |
| --- | --- | --- | --- | --- | --- |
| **Sample type** | **Number of samples** | **No. of *E. Coli* isolated** | **No. of samples positive for atleast one virulence gene** | **Virulence genes** | **Frequency** |
| Ghee | 100 | 10 | 03 | *stx1* | 01 |
|  |  |  |  | *stx1+stx2* | 01 |
|  |  |  |  | *stx1+stx2+eaeA+hlyA* | 01 |
| Paneer | 120 | 30 | 08 | *stx1+eaeA* | 01 |
|  |  |  |  | *stx1* | 02 |
|  |  |  |  | *eaeA+hlyA* | 01 |
|  |  |  |  | *stx1+stx2+eaeA* | 01 |
|  |  |  |  | *stx1+stx2* | 02 |
|  |  |  |  | *eaeA* | 01 |
| Lassi | 100 | 32 | 10 | *stx1* | 02 |
|  |  |  |  | *stx1+stx2* | 03 |
|  |  |  |  | *stx1+eaeA* | 02 |
|  |  |  |  | *stx2+hlyA+eaeA* | 01 |
|  |  |  |  | *stx1+hlyA* | 01 |
|  |  |  |  | *eaeA* | 01 |
| Dahi | 100 | 43 | 12 | *hlyA+eaeA* | 02 |
|  |  |  |  | *stx2+eaeA* | 01 |
|  |  |  |  | *stx1* | 04 |
|  |  |  |  | *Stx1+stx2* | 03 |
|  |  |  |  | *Stx1+hlyA* | 02 |
| Total | 420 | 115 | 33 |  | 33 |

| **Table S5: Virulence gene profiles of *E. coli* isolated from the milk samples** | | |
| --- | --- | --- |
| **S. No.** | **Serogroup** | **Number of isolates** |
| 1. | O18 | 10 |
| 2. | O126 | 04 |
| 3. | O17 | 04 |
| 4. | O120 | 05 |
| 5. | O111 | 03 |
| 6. | Untypeable | 03 |
| 7. | O26 | 02 |
| 8. | O134 | 02 |
| 9. | O119 | 02 |
| 11. | O5 | 02 |
| 12. | O20 | 02 |
| 13. | O135 | 01 |
| 14. | O63 | 01 |
| 15. | O157 | 01 |
| 16. | O101 | 01 |
| 17. | O64 | 01 |
| 18. | O88 | 01 |

| **Table S6: Virulence gene profiles of *E. coli* isolated fom the milk products samples** | | | |
| --- | --- | --- | --- |
| **S. No.** | **Serogroup** | **Type of sample** | **Number of isolates** |
| 1. | O18 | Ghee, dahi, paneer, lassi-5 | 08 |
| 2. | O126 | Dahi, paneer, lassi | 03 |
| 3. | O17 | Dahi-2, lassi | 03 |
| 4. | O120 | Paneer, dahi | 02 |
| 5. | O111 | Dahi-2, paneer-2 | 04 |
| 6. | Untypeable | Dahi, paneer, lassi | 03 |
| 7. | O26 | Dahi | 01 |
| 8. | O134 | Ghee | 01 |
| 9. | O119 | paneer | 01 |
| 10. | O5 | lassi | 01 |
| 11. | O20 | ghee | 01 |
| 12. | O76 | Dahi | 01 |
| 13. | O84 | Lassi | 01 |
| 14. | O11 | Paneer | 01 |
| 15. | O86 | Dahi | 01 |
| 16. | O121 | Dahi | 01 |

| **Table S7: Details of optical density (OD) obtained after staining Vero cells and visualizing in ELISA reader** | | |
| --- | --- | --- |
| **Well No.** | **Dilution** | **ELISA reading** |
| 1 | Without toxin | 0.192 |
| 2 | 1:5 | 0.144 |
| 3 | 1:10 | 0.179 |
| 4 | 1:20 | 0.181 |
| 5 | 1:40 | 0.186 |
| 6 | 1:80 | 0.191 |
| 7 | 1:5 | 0.142 |
| 8 | 1:10 | 0.161 |
| 9 | 1:20 | 0.178 |
| 10 | 1:40 | 0.182 |
| 11 | 1:80 | 0.190 |
| 12 | Undiluted toxin | 0.121 |

| **Table S8.Antimicrobial sensitivity and resistance pattern of STEC and EPEC isolates from milk** | | | | | |
| --- | --- | --- | --- | --- | --- |
| **S. No.** | **Antimicrobial agent** | **Concentration per disc in µg** | **Sensitive** | **Intermediate** | **Resistant** |
| **1.** | Amoxicillin | 30 | 14 | 17 | 14 |
| **2.** | Enrofloxacin | 10 | 07 | 07 | 31 |
| **3.** | Oxytetracycline | 30 | 06 | 09 | 35 |
| **4.** | Sulphadiazine | 100 | 05 | 02 | 38 |
| **5.** | Tetracycline | 10 | 07 | 02 | 36 |
| **6.** | Vancomycin | 10 | 13 | 20 | 12 |
| **7.** | Streptomycin | 10 | 12 | 21 | 12 |
| **8.** | Cefixime | 5 | 18 | 19 | 08 |
| **9.** | Ceftazidime | 30 | 21 | 17 | 07 |
| **10.** | Ceftriaxone | 30 | 18 | 17 | 10 |
| **11.** | Chloramphenicol | 30 | 19 | 07 | 19 |
| **12.** | Co-trimoxazole | 10 | 14 | 21 | 10 |
| **13.** | Gentamicin | 120 | 36 | 04 | 05 |
| **14.** | Imipenem | 10 | 40 | 05 | 00 |
| **15.** | Nalidixic acid | 30 | 39 | 04 | 03 |
| **16.** | Ampicillin | 10 | 05 | 01 | 39 |
| **17.** | Azithromycin | 15 | 17 | 18 | 10 |
| **18.** | Erythromycin | 30 | 06 | 20 | 19 |
| **19.** | Cephalothin | 30 | 03 | 04 | 38 |

**
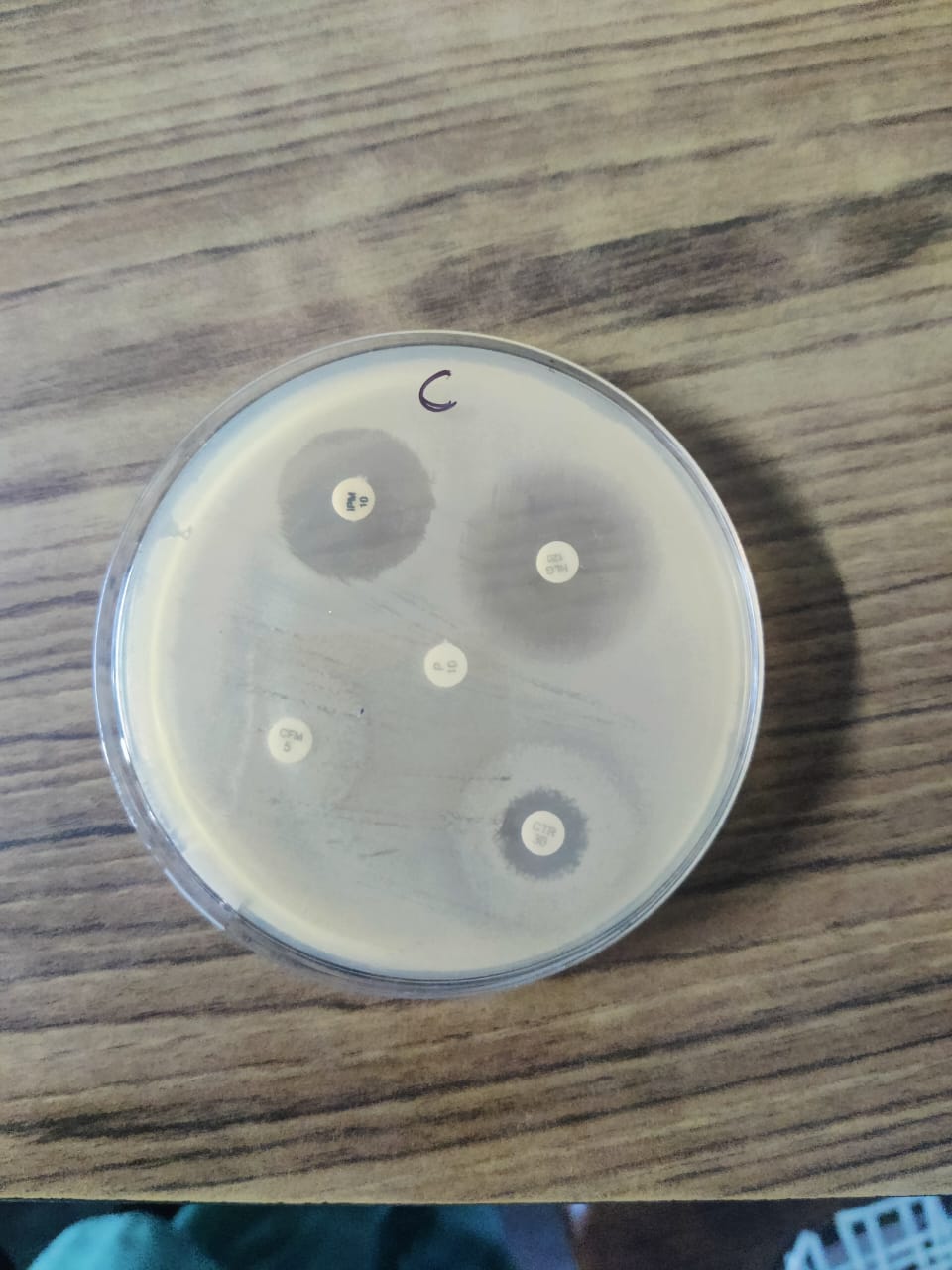
**

**Fig S4: Antibiotic susceptibility test result**

| **Table S9.Antimicrobial sensitivity and resistance pattern of STEC and EPEC isolates from milk products (Ghee)** | | | | | |
| --- | --- | --- | --- | --- | --- |
| **Sl. No.** | **Antimicrobial agent** | **Concentration per disc in µg** | **Sensitive** | **Intermediate** | **Resistant** |
| **1.** | Amoxicillin | 30 | 01 | - | 02 |
| **2.** | Enrofloxacin | 10 | 01 | 01 | 01 |
| **3.** | Oxytetracycline | 30 | 00 | - | 03 |
| **4.** | Sulphadiazine | 100 | 00 | - | 03 |
| **5.** | Tetracycline | 10 | 00 | - | 03 |
| **6.** | Vancomycin | 10 | 01 | 02 | 00 |
| **7.** | Streptomycin | 10 | 00 | 03 | 00 |
| **8.** | Cefixime | 5 | 01 | - | 02 |
| **9.** | Ceftazidime | 30 | 01 | - | 02 |
| **10.** | Ceftriaxone | 30 | 01 | - | 02 |
| **11.** | Chloramphenicol | 30 | 02 | 01 | 00 |
| **12.** | Co-trimoxazole | 10 | 03 | - | 00 |
| **13.** | Gentamicin | 120 | 03 | - | 00 |
| **14.** | Imipenem | 10 | 03 | - | 00 |
| **15.** | Nalidixic acid | 30 | 03 | - | 00 |
| **16.** | Ampicillin | 10 | 00 | - | 03 |
| **17.** | Azithromycin | 15 | 01 | 02 | 00 |
| **18.** | Erythromycin | 30 | 03 | - | 00 |
| **19.** | Cephalothin | 30 | 00 | - | 03 |

| **Table S10.Antimicrobial sensitivity and resistance pattern of STEC and EPEC isolates from milk products (Paneer)** | | | | | |
| --- | --- | --- | --- | --- | --- |
| **Serial no.** | **Antimicrobial agent** | **Concentration per disc in µg** | **Sensitive** | **Intermediate** | **Resistant** |
| 1. | Amoxicillin | 30 | 02 | - | 06 |
| 2. | Enrofloxacin | 10 | 03 | - | 05 |
| 3. | Oxytetracycline | 30 | 00 | - | 08 |
| 4. | Sulphadiazine | 100 | 02 | 04 | 02 |
| 5. | Tetracycline | 10 | 00 | - | 08 |
| 6. | Vancomycin | 10 | 01 | - | 07 |
| 7. | Streptomycin | 10 | 02 | 01 | 05 |
| 8. | Cefixime | 5 | 04 | - | 04 |
| 9. | Ceftazidime | 30 | 05 | - | 03 |
| 10. | Ceftriaxone | 30 | 03 | - | 05 |
| 11. | Chloramphenicol | 30 | 02 | 05 | 01 |
| 12. | Co-trimoxazole | 10 | 02 | 03 | 03 |
| 13. | Gentamicin | 120 | 08 | - | 00 |
| 14. | Imipenem | 10 | 08 | - | 00 |
| 15. | Nalidixic acid | 30 | 08 | - | 00 |
| 17. | Ampicillin | 10 | 01 | - | 07 |
| 18. | Azithromycin | 15 | 02 | 04 | 02 |
| 19. | Erythromycin | 30 | 02 | 06 | 00 |
| 20. | Cephalothin | 30 | 00 | - | 08 |

| **Table S11.Antimicrobial sensitivity and resistance pattern of STEC and EPEC isolates from milk products (Lassi)** | | | | | |
| --- | --- | --- | --- | --- | --- |
| **S. No.** | **Antimicrobial agent** | **Concentration per disc in µg** | **Sensitive** | **Intermediate** | **Resistant** |
| 1. | Amoxicillin | 30 | 03 | - | 07 |
| 2. | Enrofloxacin | 10 | 04 | - | 06 |
| 3. | Oxytetracycline | 30 | 00 | - | 10 |
| 4. | Sulphadiazine | 100 | 02 | - | 08 |
| 5. | Tetracycline | 10 | 00 | - | 10 |
| 6. | Vancomycin | 10 | 03 | - | 07 |
| 7. | Streptomycin | 10 | 04 | 05 | 01 |
| 8. | Cefixime | 5 | 01 | 08 | 01 |
| 9. | Ceftazidime | 30 | 03 | 02 | 05 |
| 10. | Ceftriaxone | 30 | 06 | - | 04 |
| 11. | Chloramphenicol | 30 | 03 | 01 | 06 |
| 12. | Co-trimoxazole | 10 | 02 | 01 | 07 |
| 13. | Gentamicin | 120 | 10 | - | 00 |
| 14. | Imipenem | 10 | 09 | - | 01 |
| 15. | Nalidixic acid | 30 | 10 | - | 00 |
| 16. | Ampicillin | 10 | 00 | - | 10 |
| 17. | Azithromycin | 15 | 03 | - | 07 |
| 18. | Erythromycin | 30 | 02 | 03 | 05 |
| 19. | Cephalothin | 30 | 03 | - | 07 |

| **Table S12. Antimicrobial sensitivity and resistance pattern of STEC and EPEC isolates from milk products (Dahi)** | | | | | |
| --- | --- | --- | --- | --- | --- |
| **Sl. No.** | **Antimicrobial agent** | **Concentration per disc in µg** | **Sensitive** | **Intermediate** | **Resistant** |
| **1.** | Amoxicillin | 30 | 05 | 04 | 03 |
| **2.** | Enrofloxacin | 10 | 06 | - | 06 |
| **3.** | Oxytetracycline | 30 | 00 | - | 12 |
| **4.** | Sulphadiazine | 100 | 00 | - | 12 |
| **5.** | Tetracycline | 10 | 00 | - | 12 |
| **6.** | Vancomycin | 10 | 04 | - | 08 |
| **7.** | Streptomycin | 10 | 03 | - | 09 |
| **8.** | Cefixime | 5 | 05 | - | 07 |
| **9.** | Ceftazidime | 30 | 06 | - | 06 |
| **10.** | Ceftriaxone | 30 | 07 | - | 05 |
| **11.** | Chloramphenicol | 30 | 07 | - | 05 |
| **12.** | Co-trimoxazole | 10 | 06 | - | 06 |
| **13.** | Gentamicin | 120 | 10 | 02 | 00 |
| **14.** | Imipenem | 10 | 11 | 01 | 00 |
| **15.** | Nalidixic acid | 30 | 09 | 00 | 03 |
| **16.** | Ampicillin | 10 | 02 | 00 | 10 |
| **17.** | Azithromycin | 15 | 01 | - | 11 |
| **18.** | Erythromycin | 30 | 03 | - | 09 |
| **19.** | Cephalothin | 30 | 00 | - | 12 |

**
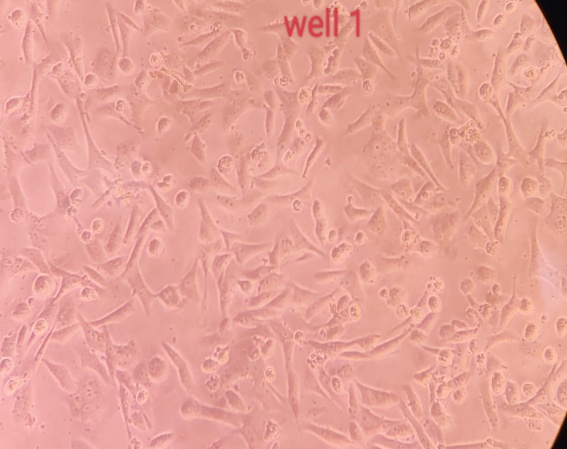

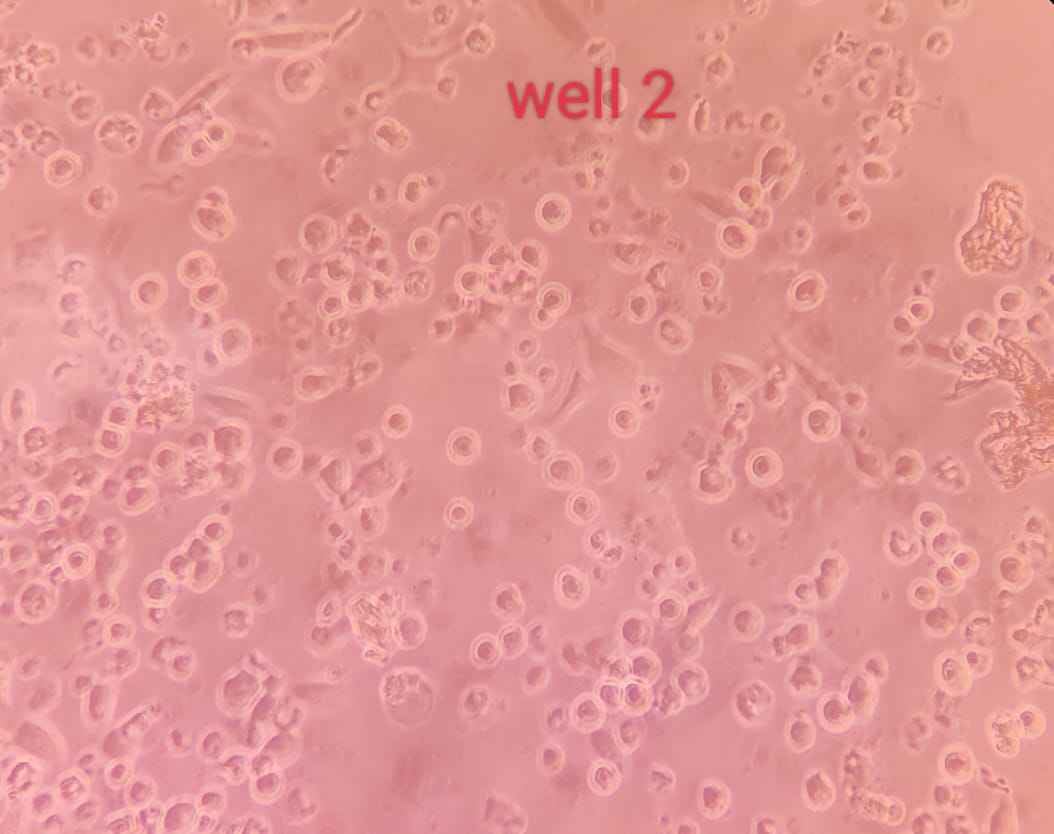
**

| **Fig S5a : Vero cells after 24 hr of incubation (400X)** |  | **Fig S5b: Verocells after 24hrs. incubation with 1:5 dilution toxin showing rounding of cells (400X)** |
| --- | --- | --- |

**
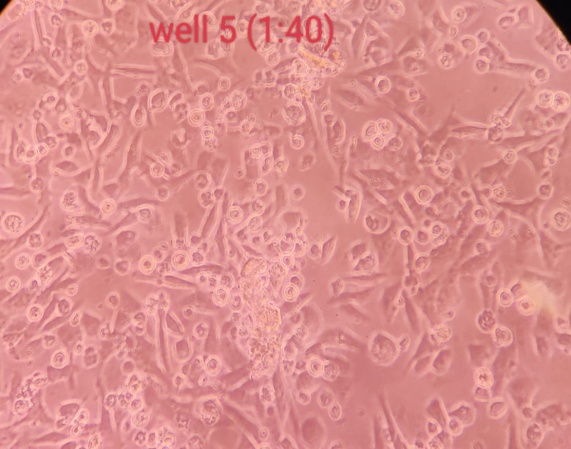
**

| **Fig S5c: Verocells after 24 hrs. incubation with (1:40) dilution toxin (400X)** |  |  |
| --- | --- | --- |

**
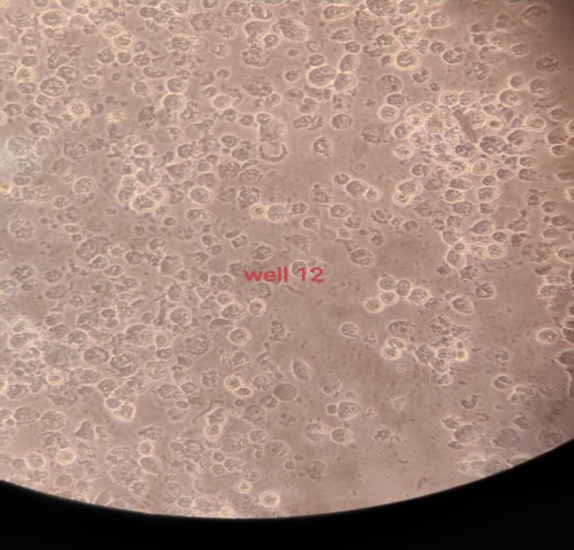

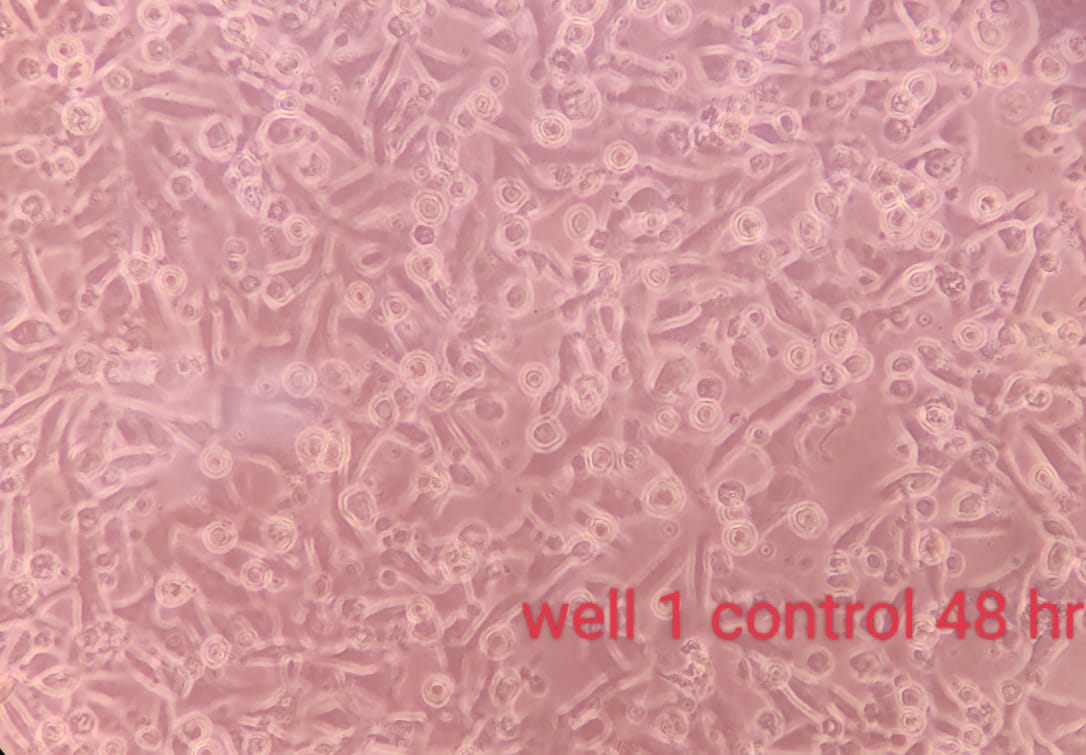
**

| **Fig S5d: Incubation of Vero cells in pure toxin resulting in cell death after 24hrs.(400X)** |  | **Fig S5e: Control well containing only Vero cells after 48hrs. (400X)** |
| --- | --- | --- |

**
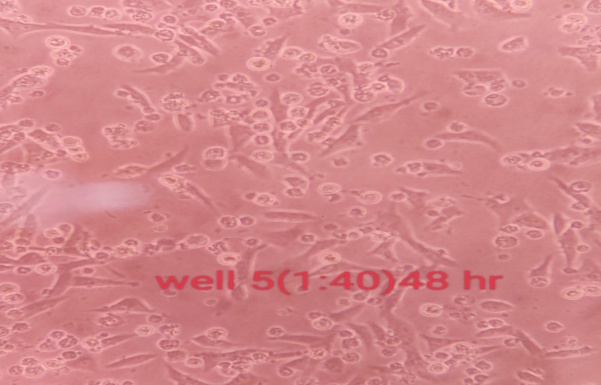
**

**Fig S5f: Incubation of Vero cells in (1:40) dilution toxin resulting in cell roundening and syncytia formation after 48hrs (400X)**

**
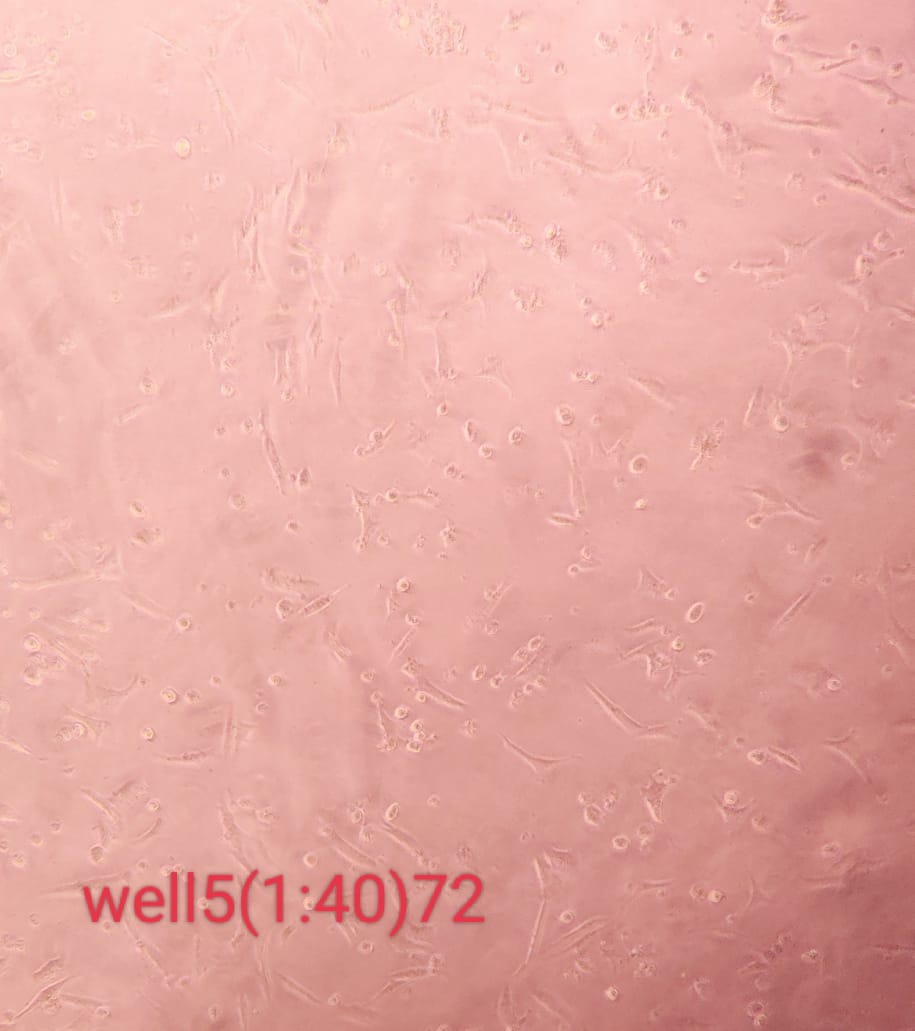
**

**Fig S5g: Incubation of Vero cells in (1:40) dilution toxin after 72hrs (400X)**

**
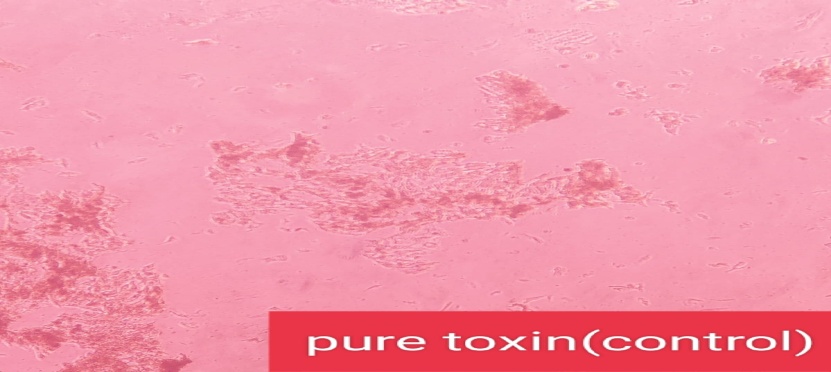
**

| **Fig S5h: Incubation of Vero cells in pure toxin after 72hrs cell clumping after death is seen (400X)** |  |
| --- | --- |

**
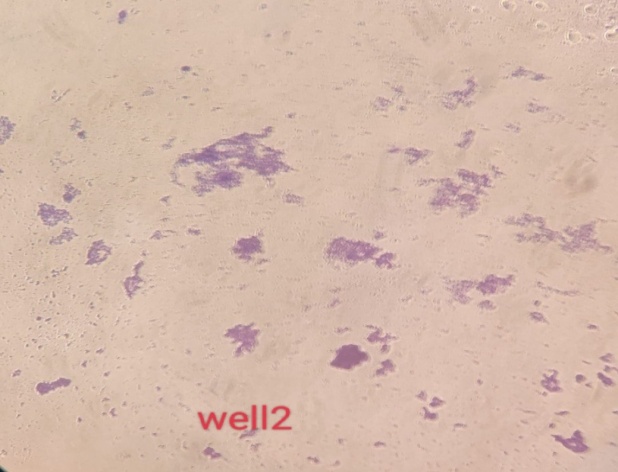
**

**Fig S5i: Staining of Vero cells with (1:5) dilution Shiga toxin control by 0.13% crystal violet stain after 72hrs (400X)**

**
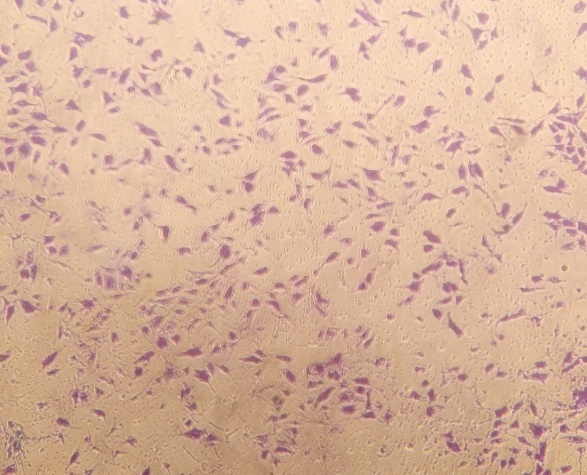
**

| **Fig S5j: Staining of Vero cells with (1:40) dilution Shiga toxin control by 0.13% crystal violet stain after 72hrs (400X)** |  |  |
| --- | --- | --- |
