# Supplementary material for: Molecular detection, serotyping, cytotoxicity, and antimicrobial resistance of STEC and EPEC isolated from milk and milk products in northern India
Source: Front Microbiol. 2026 Feb 18;17:1748367. doi: 10.3389/fmicb.2026.1748367 (PMC12957279; doi:10.3389/fmicb.2026.1748367)
Supplement: Supplementary file 2 [file Data_Sheet_1.PDF]

Supplementary Tables and Figures

Table S1. Sample collection from different locations in Uttarakhand		
**S. No.**	**Sample type**	**Number of samples**
1.	Raw milk	260
2.	Ghee	100
3.	Paneer	120
4.	Lassi	100
5.	Dahi	100
	Total	680

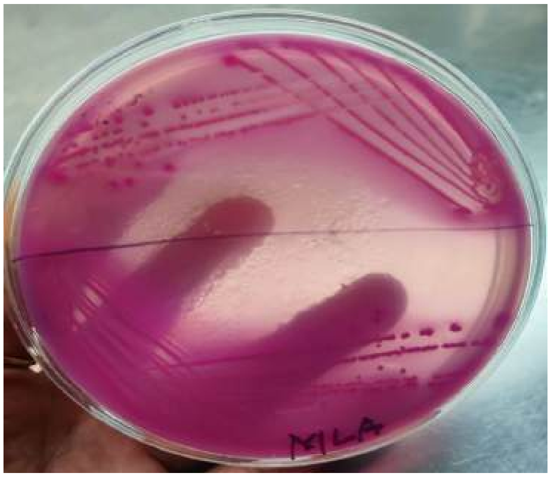

Fig S1: Lactose fermenting pink coloured colonies of *E. coli* on MacConkey agar Fig

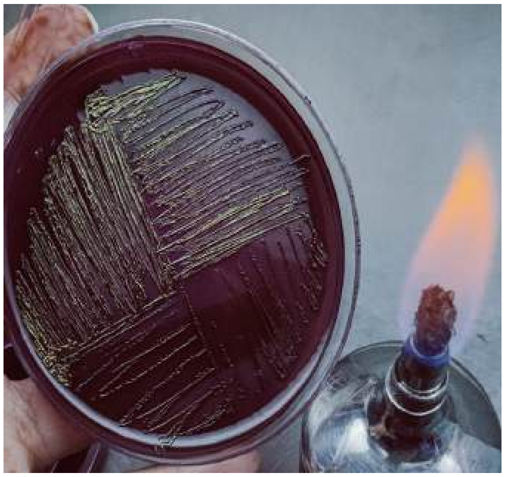

Fig S2: Greenish metallic sheen colonies of *E. coli* on eosin methylene blue agar

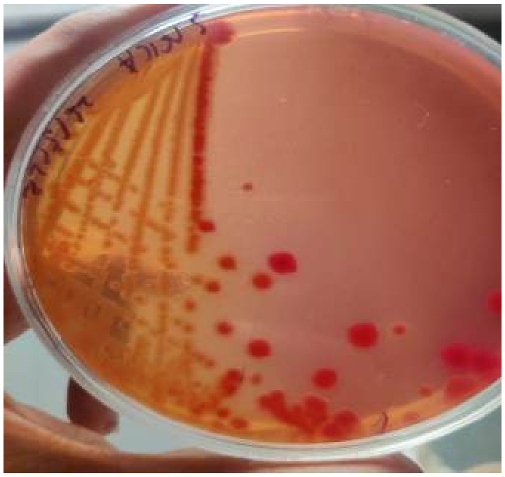

Fig S3: Pink and pale coloured colonies of *E. coli* non-O157 on SMAC agar Fig

Table S2. Antibiotics used in sensitivity testing		
**S. No.**	**Antimicrobial reagent**	**Concentration per disc**
1.	Gentamicin(HLG)	120 µg
2.	Ceftriaxone(CTR)	30 µg
3.	Cefixime(CFM)	5 µg
4.	Cephalothin(CEP)	30 µg
5.	Ceftazidime(CAZ)	30 µg
6.	Chloramphenicol(C)	30 µg
7.	Imipenem(IPM)	10 µg
8.	Enrofloxacin(EX)	10 µg
9.	Clotrimazole(CC)	10 µg
10.	Erythromycin(E)	30 µg
11.	Tetracycline(TE)	10 µg
12.	Nalidixic Acid(NA)	30 µg
13.	Azithromycin(AZM)	15 µg
14.	Sulphadiazine(SZ)	100 µg
15.	Oxytetracycline(O)	30 µg
16.	Amoxycillin(AMX)	30 µg
17.	Ampicillin(AMP)	10 µg
18.	Streptomycin(S)	10 µg
19.	Vancomycin(VA)	10 µg

Table S3.Distribution of the virulence genes in *E. coli* isolated from the milk samples					
**No. of milk samples collected**	**Number of *E. coli* isolated**	**No. of *E. coli* isolates positive for atleast one virulence gene**	**Virulence gene recovered**	**No. of positive isolates recovered from**	**Percentage prevalence**
260	81	45	*stx1*	07	8.64
			stx2	03	3.70
			HlyA	04	4.94
			eaeA	04	4.94
			stx1+stx2	03	3.70
			stx1+eaeA	06	7.41
			Stx1+hlyA	01	1.23
			stx1+eaeA+hlyA	01	1.23
			stx2+eaeA	02	2.47

			stx2+hlyA	05	6.17
			eaeA+hlyA	03	3.70
			stx1+stx2+eaeA	02	2.47
			stx1+stx2+eaeA +hlyA	04	4.94
			**Total**	45	55.56

Table S4. Distribution of virulence genes in *E. coli* isolated from the milk product samples

Sample type	Number of samples	No. of *E. Coli* isolated	No. of samples positive for atleast one virulence gene	Virulence genes	Frequency
Ghee	100	10	03	*stx1*	01
				stx1+stx2	01
				stx1+stx2+eaeA+hlyA	01
Paneer	120	30	08	*stx1+eaeA*	01
				stx1	02
				eaeA+hlyA	01
				stx1+stx2+eaeA	01
				stx1+stx2	02
				eaeA	01
Lassi	100	32	10	*stx1*	02
				stx1+stx2	03
				stx1+eaeA	02
				stx2+hlyA+eaeA	01
				stx1+hlyA	01
				eaeA	01
Dahi	100	43	12	*hlyA+eaeA*	02
				stx2+eaeA	01
				stx1	04
				Stx1+stx2	03
				Stx1+hlyA	02
Total	420	115	33		33

Table S5: Virulence gene profiles of *E. coli* isolated from the milk samples		
**S. No.**	**Serogroup**	**Number of isolates**
1.	O18	10
2.	O126	04
3.	O17	04
4.	O120	05
5.	O111	03
6.	Untypeable	03
7.	O26	02
8.	O134	02
9.	O119	02
11.	O5	02
12.	O20	02
13.	O135	01
14.	O63	01
15.	O157	01
16.	O101	01
17.	O64	01
18.	O88	01

**Table S6: Virulence gene profiles of *E. coli* isolated fom the milk products samples**			
**S. No.**	**Serogroup**	**Type of sample**	**Number of isolates**
1.	O18	Ghee, dahi, paneer, lassi-5	08
2.	O126	Dahi, paneer, lassi	03
3.	O17	Dahi-2, lassi	03
4.	O120	Paneer, dahi	02
5.	O111	Dahi-2, paneer-2	04
6.	Untypeable	Dahi, paneer, lassi	03
7.	O26	Dahi	01
8.	O134	Ghee	01
9.	O119	paneer	01
10.	O5	lassi	01
11.	O20	ghee	01
12.	O76	Dahi	01
13.	O84	Lassi	01
14.	O11	Paneer	01
15.	O86	Dahi	01
16.	O121	Dahi	01

Table S7: Details of optical density (OD) obtained after staining Vero cells and visualizing in ELISA reader		
**Well No.**	**Dilution**	**ELISA reading**
1	Without toxin	0.192
2	1:5	0.144
3	1:10	0.179
4	1:20	0.181
5	1:40	0.186
6	1:80	0.191
7	1:5	0.142
8	1:10	0.161
9	1:20	0.178
10	1:40	0.182
11	1:80	0.190
12	Undiluted toxin	0.121

Table S8.Antimicrobial sensitivity and resistance pattern of STEC and EPEC isolates from milk					
**S. No.**	**Antimicrobial agent**	**Concentration per disc in µg**	**Sensitive**	**Intermediate**	**Resistant**
**1.**	Amoxicillin	30	14	17	14
**2.**	Enrofloxacin	10	07	07	31
**3.**	Oxytetracycline	30	06	09	35
**4.**	Sulphadiazine	100	05	02	38
**5.**	Tetracycline	10	07	02	36
**6.**	Vancomycin	10	13	20	12
**7.**	Streptomycin	10	12	21	12
**8.**	Cefixime	5	18	19	08
**9.**	Ceftazidime	30	21	17	07
**10.**	Ceftriaxone	30	18	17	10
**11.**	Chloramphenicol	30	19	07	19
**12.**	Co-trimoxazole	10	14	21	10
**13.**	Gentamicin	120	36	04	05
**14.**	Imipenem	10	40	05	00
**15.**	Nalidixic acid	30	39	04	03
**16.**	Ampicillin	10	05	01	39
**17.**	Azithromycin	15	17	18	10
**18.**	Erythromycin	30	06	20	19
**19.**	Cephalothin	30	03	04	38

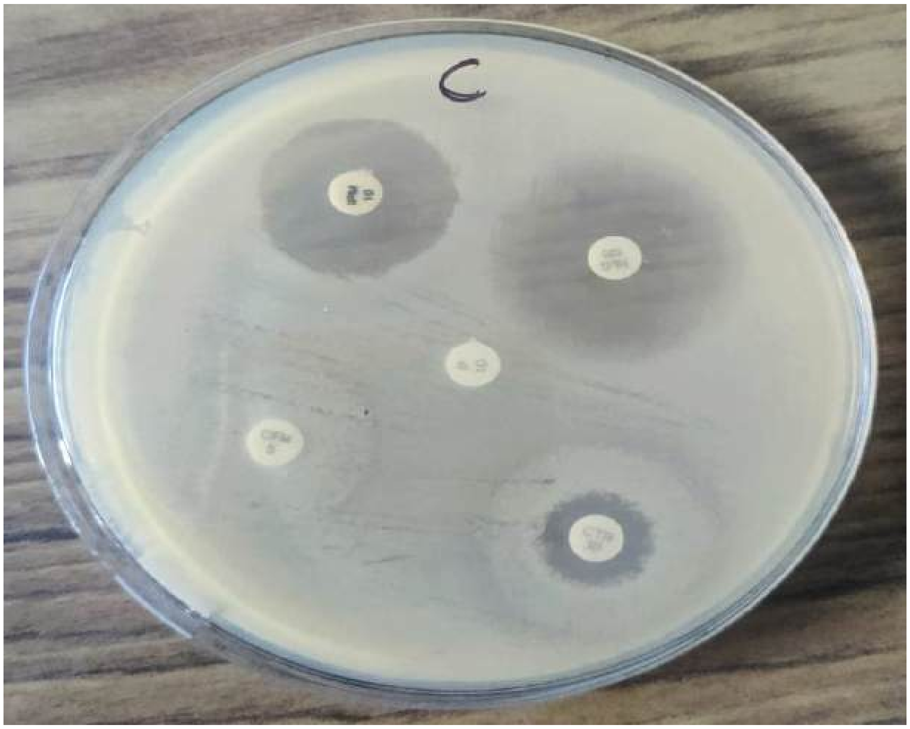

Fig S4: Antibiotic susceptibility test result

**Table S9.Antimicrobial sensitivity and resistance pattern of STEC and EPEC isolates from milk products (Ghee)**					
**Sl. No.**	**Antimicrobial agent**	**Concentration per disc in µg**	**Sensitive**	**Intermediate**	**Resistant**
**1.**	Amoxicillin	30	01	-	02
**2.**	Enrofloxacin	10	01	01	01
**3.**	Oxytetracycline	30	00	-	03
**4.**	Sulphadiazine	100	00	-	03
**5.**	Tetracycline	10	00	-	03
**6.**	Vancomycin	10	01	02	00
**7.**	Streptomycin	10	00	03	00
**8.**	Cefixime	5	01	-	02
**9.**	Ceftazidime	30	01	-	02
**10.**	Ceftriaxone	30	01	-	02
**11.**	Chloramphenicol	30	02	01	00
**12.**	Co-trimoxazole	10	03	-	00
**13.**	Gentamicin	120	03	-	00
**14.**	Imipenem	10	03	-	00
**15.**	Nalidixic acid	30	03	-	00
**16.**	Ampicillin	10	00	-	03
**17.**	Azithromycin	15	01	02	00
**18.**	Erythromycin	30	03	-	00
**19.**	Cephalothin	30	00	-	03

**Table S10.Antimicrobial sensitivity and resistance pattern of STEC and EPEC isolates from milk products (Paneer)**					
**Serial no.**	**Antimicrobial agent**	**Concentration per disc in µg**	**Sensitive**	**Intermediate**	**Resistant**
1.	Amoxicillin	30	02	-	06
2.	Enrofloxacin	10	03	-	05
3.	Oxytetracycline	30	00	-	08
4.	Sulphadiazine	100	02	04	02
5.	Tetracycline	10	00	-	08
6.	Vancomycin	10	01	-	07
7.	Streptomycin	10	02	01	05
8.	Cefixime	5	04	-	04
9.	Ceftazidime	30	05	-	03
10.	Ceftriaxone	30	03	-	05
11.	Chloramphenicol	30	02	05	01
12.	Co-trimoxazole	10	02	03	03
13.	Gentamicin	120	08	-	00
14.	Imipenem	10	08	-	00
15.	Nalidixic acid	30	08	-	00
17.	Ampicillin	10	01	-	07
18.	Azithromycin	15	02	04	02
19.	Erythromycin	30	02	06	00
20.	Cephalothin	30	00	-	08

**Table S11.Antimicrobial sensitivity and resistance pattern of STEC and EPEC isolates from milk products (Lassi)**					
**S. No.**	**Antimicrobial agent**	**Concentration per disc in µg**	**Sensitive**	**Intermediate**	**Resistant**
1.	Amoxicillin	30	03	-	07
2.	Enrofloxacin	10	04	-	06
3.	Oxytetracycline	30	00	-	10
4.	Sulphadiazine	100	02	-	08
5.	Tetracycline	10	00	-	10
6.	Vancomycin	10	03	-	07
7.	Streptomycin	10	04	05	01
8.	Cefixime	5	01	08	01
9.	Ceftazidime	30	03	02	05
10.	Ceftriaxone	30	06	-	04
11.	Chloramphenicol	30	03	01	06
12.	Co-trimoxazole	10	02	01	07
13.	Gentamicin	120	10	-	00
14.	Imipenem	10	09	-	01
15.	Nalidixic acid	30	10	-	00
16.	Ampicillin	10	00	-	10
17.	Azithromycin	15	03	-	07
18.	Erythromycin	30	02	03	05
19.	Cephalothin	30	03	-	07

**Table S12. Antimicrobial sensitivity and resistance pattern of STEC and EPEC isolates from milk products (Dahi)**					
**Sl. No.**	**Antimicrobial agent**	**Concentration per disc in µg**	**Sensitive**	**Intermediate**	**Resistant**
**1.**	Amoxicillin	30	05	04	03
**2.**	Enrofloxacin	10	06	-	06
**3.**	Oxytetracycline	30	00	-	12
**4.**	Sulphadiazine	100	00	-	12
**5.**	Tetracycline	10	00	-	12
**6.**	Vancomycin	10	04	-	08
**7.**	Streptomycin	10	03	-	09
**8.**	Cefixime	5	05	-	07
**9.**	Ceftazidime	30	06	-	06
**10.**	Ceftriaxone	30	07	-	05
**11.**	Chloramphenicol	30	07	-	05
**12.**	Co-trimoxazole	10	06	-	06
**13.**	Gentamicin	120	10	02	00
**14.**	Imipenem	10	11	01	00
**15.**	Nalidixic acid	30	09	00	03
**16.**	Ampicillin	10	02	00	10
**17.**	Azithromycin	15	01	-	11
**18.**	Erythromycin	30	03	-	09
**19.**	Cephalothin	30	00	-	12

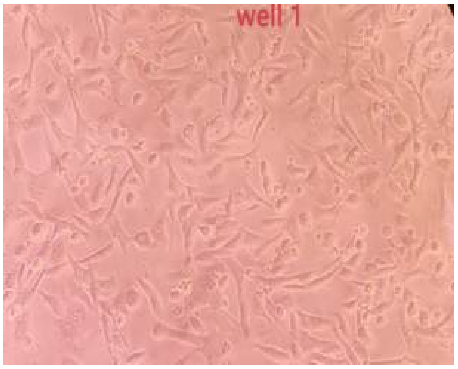

Fig S5a : Vero cells after 24 hr of incubation (400X)

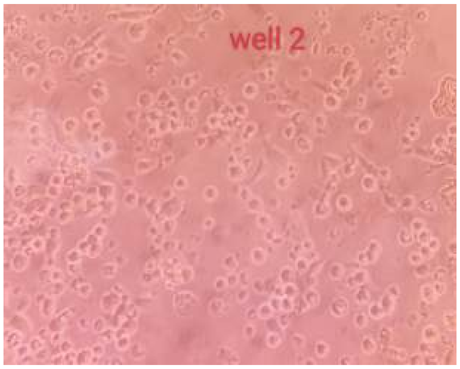

Fig S5b: Verocells after 24hrs. incubation with 1:5 dilution toxin showing rounding of cells (400X)

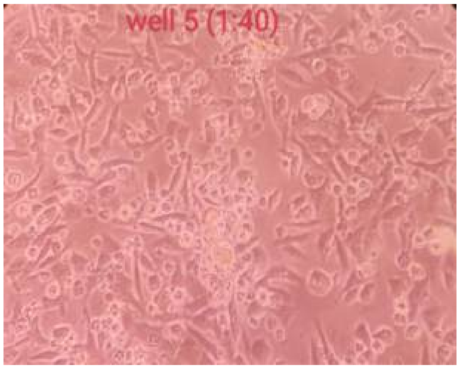

Fig S5c: Verocells after 24 hrs. incubation with (1:40) dilution toxin (400X)

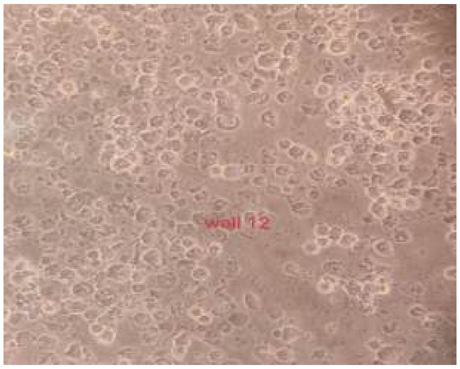

Fig S5d: Incubation of Vero cells in pure toxin resulting in cell death after 24hrs.(400X)

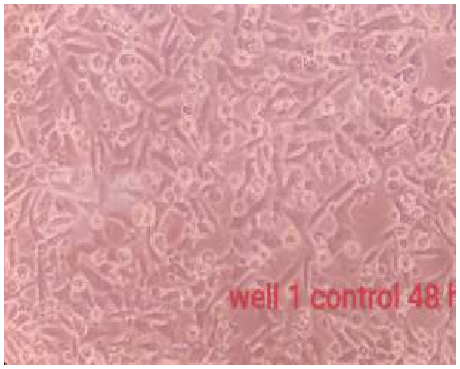

Fig S5e: Control well containing only Vero cells after 48hrs. (400X)

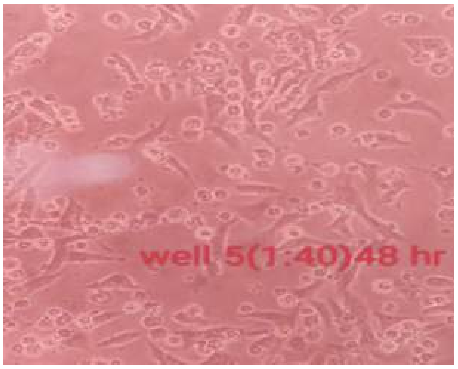

Fig S5f: Incubation of Vero cells in (1:40) dilution toxin resulting in cell roundening and syncytia formation after 48hrs (400X)

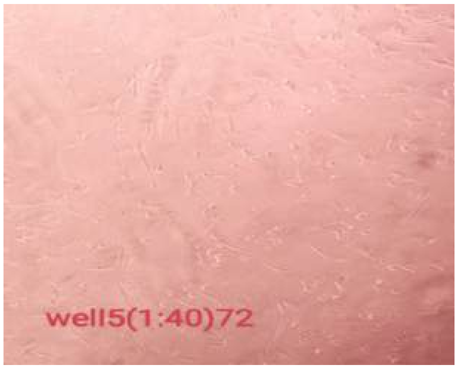

Fig S5g: Incubation of Vero cells in (1:40) dilution toxin after 72hrs (400X)

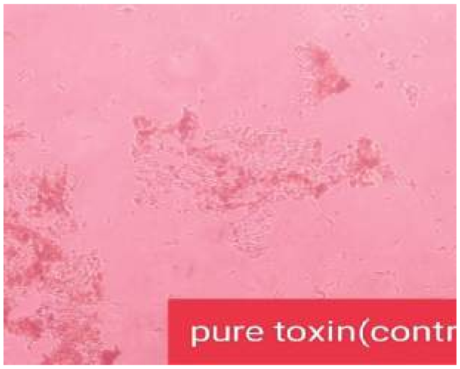

Fig S5h: Incubation of Vero cells in pure toxin after 72hrs cell clumping after death is seen (400X)

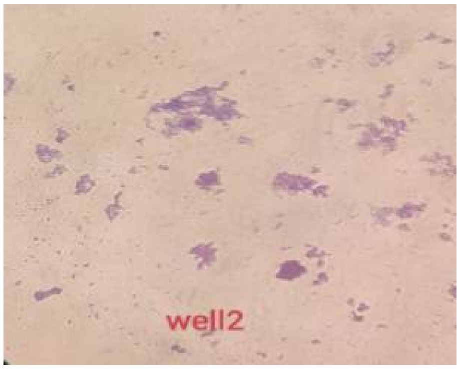

Fig S5i: Staining of Vero cells with (1:5) dilution toxin by 0.13% crystal violet stain after 72hrs.(400X)

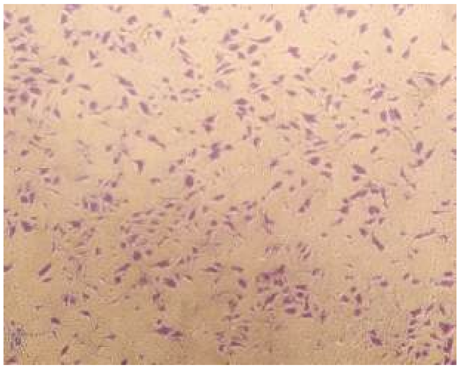

Fig S5j: Staining of Vero cells with (1:40) dilution toxin by 0.13% crystal violet stain after 72hrs.(400X)
